# Assessment of Habitat Suitability Is Affected by Plant-Soil Feedback: Comparison of Field and Garden Experiment

**DOI:** 10.1371/journal.pone.0157800

**Published:** 2016-06-23

**Authors:** Lucie Hemrová, Jana Knappová, Zuzana Münzbergová

**Affiliations:** 1 Department of Botany, Faculty of Science, Charles University in Prague, Prague, Czech Republic; 2 Institute of Botany, Academy of Sciences of the Czech Republic, Průhonice, Czech Republic; Technical University in Zvolen, SLOVAKIA

## Abstract

**Background:**

Field translocation experiments (i.e., the introduction of seeds or seedlings of different species into different localities) are commonly used to study habitat associations of species, as well as factors limiting species distributions and local abundances. Species planted or sown in sites where they naturally occur are expected to perform better or equally well compared to sites at which they do not occur or are rare. This, however, contrasts with the predictions of the Janzen-Connell hypothesis and commonly reported intraspecific negative plant-soil feedback. The few previous studies indicating poorer performance of plants at sites where they naturally occur did not explore the mechanisms behind this pattern.

**Aims and Methods:**

In this study, we used field translocation experiments established using both seeds and seedlings to study the determinants of local abundance of four dominant species in grasslands. To explore the possible effects of intraspecific negative plant-soil feedback on our results, we tested the effect of local species abundance on the performance of the plants in the field experiment. In addition, we set up a garden experiment to explore the intensity of intraspecific as well as interspecific feedback between the dominants used in the experiment.

**Key Results:**

In some cases, the distribution and local abundances of the species were partly driven by habitat conditions at the sites, and species performed better at their own sites. However, the prevailing pattern was that the local dominants performed worse at sites where they naturally occur than at any other sites. Moreover, the success of plants in the field experiment was lower in the case of higher intraspecific abundance prior to experimental setup. In the garden feedback experiment, two of the species performed significantly worse in soils conditioned by their species than in soils conditioned by the other species. In addition, the performance of the plants was significantly correlated between the two experiments, suggesting that plant-soil feedback is a likely explanation of the patterns observed in the field.

**Conclusions:**

All of the results indicate that intraspecific negative plant-soil feedback, either biotic or abiotic, may be a key factor determining the performance of the plants in our field translocation experiment. The possible effects of negative feedback should thus be considered when evaluating results of translocation experiments in future studies.

## Introduction

Understanding the factors determining the distribution of species in the landscape as well as their local abundances is one of the most important topics in current ecology ([1–3]). The association of species with specific environmental conditions is commonly studied using translocation (transplant) experiments [4]. In these experiments, species are typically planted or sown to compare performance or other attributes among various sites at which they naturally do and do not occur. For example, many recent studies have used such an approach to explore the intensity of edaphic associations of different species in tropical rainforests (e.g., [5–10]). Similarly, habitat associations have also been explored using transplant experiments in temperate forest trees [11] and herbs [12]. The same experimental design, i.e., sowing or planting plants at occupied and unoccupied sites, is also commonly used to study the importance of habitat vs. dispersal limitations of species at a range of spatial scales, with a majority of these studies being performed in grassland ecosystems (e.g., [13–20]).

In all of the above experiments, it is expected that species planted or sown at sites where they naturally occur will perform better or equally well compared to the sites at which they do not occur or are rare. Better performance at sites naturally occupied by the species is taken as support for the idea that these species show strong habitat associations and are thus mainly habitat-limited. Equal performance at all sites, on the other hand, is considered to demonstrate that the species are primarily dispersal-limited [21].

While most of the above cited experiments found that the species in question perform better or equally well at occupied sites than at unoccupied sites, exceptions to this pattern have been found in a range of studies of some species and experimental areas (e.g., [5], [6], [13], [14], [22]), indicating that other results, i.e., lower performance at occupied than at unoccupied sites, might occur. None of these studies, however, extensively explored the possible explanations for such a pattern.

Lower performance at occupied compared to unoccupied sites can theoretically be expected to occur based on the Janzen-Connell hypothesis ([23], [24]). This hypothesis suggests that, due to the existence of specialist natural enemies, species are more likely to recruit and establish under heterospecifics than under conspecifics, leading to the maintenance of high diversity in natural communities (see tests in e.g., [25–27]). Recently, similar effects have also been investigated in terms of plant-soil feedback (e.g., [28], [29]). Negative plant-soil feedback can be viewed as one possible mechanism responsible for the Janzen-Connell effect, promoting species coexistence and diversity by decreasing the local abundance of species at sites where they previously occurred, leading to their higher abundances in other areas [28]. Indeed, much literature supports the expectation that local species could perform worse at their home sites than at other sites due to the accumulation of species-specific pathogens and/or predators, or due to the species-specific use of soil nutrients (e.g., [30–32]). This possibility is, however, often not considered in transplant experiments designed to study the determinants of species distributions and local abundances. This can lead to incorrect conclusions regarding habitat associations of the species in question.

In this study, we used a field translocation experiment that was established using both seeds and seedlings to study the determinants of local abundance for four dominant species in grasslands. Based on the above evidence, it may be expected that intraspecific plant-soil feedback may be an important factor affecting the results of our experiment. More specifically, it can be expected that intraspecific feedback will be stronger in places where the species of interest are dominant in comparison with localities where they are in a subordinate position or absent. To explore this, we tested the effect of local species abundance (measured on a continuous scale) on the performance of the plants in the field translocation experiment. In addition, we set up an additional two-phase garden experiment to explore the intensity of intraspecific and interspecific feedback between the dominant species used in the field experiment. Specifically, we attempted to answer the following questions: 1) Are there any differences in the abiotic conditions between the localities dominated by the different species? 2) Does the performance of plants transplanted to different localities differ between localities with a different dominant? 3) What are the effects of the dominants on the soil chemical composition in the garden experiment? 4) Is plant performance in the second phase of the garden experiment affected by the species cultivated in the soil in the first phase? 5) Do the results of the field and garden experiments differ between early and later stages of plant development? 6) Is there any congruence between the results from the field and garden experiments?

## Methods

### Model species

We used four different dominant plant species of dry grasslands in northern Bohemia, Czech Republic, Europe [33], as model species. They include *Anthericum ramosum* L. (Liliaceae), *Brachypodium pinnatum* (L.) BEAUV. (Poaceae), *Bromus erectus* Huds. (Poaceae) and *Inula salicina* L. (Asteraceae) [34]. All of these species are long-lived, clonally growing perennials with a wide distribution across Europe [34]. A comparison of specific life history traits and habitat requirements of these species is given in Table 1. Most of the data given in Table 1 come from [35]. Data on the importance of habitat conditions are based on [33]. Data on niche width are based on the approach of ([36]) and were calculated in Knappová and Münzbergová [37]. Data on field germinability and field competitiveness were assessed based on data from seed sowing experiment on abandoned fields in the area ([20]).

**Table 1:** Characteristics of the target species. Sources of the data are given in the methods. EIV stands for Ellenberg indicator values.

	*A*. *ramosum*	*B*. *pinnatum*	*B*. *erectus*	*I*. *salicina*
Terminal velocity (m/s)	2.81	3.25	2.35	0.26
Endozoochory (%)^1^	43.78	69.56	74.22	43.56
Exozoochory (%)^2^	2.75	12.25	8.25	35.00
Seed weight (g)	2.49	1.78	4.93	0.20
Plant height (m)	0.64	0.78	0.81	0.42
Start of flowering (month)	6	6	5	7
Duration of flowering (month)	3	2	5	2
Specific leaf area	23.50	22.75	16.46	26.82
EIV Light	7	6	8	8
EIV Temperature	5	5	5	6
EIV Continentality	4	5	2	5
EIV Moisture	3	4	3	6
EIV pH	7	7	8	9
EIV Nutrients	3	4	3	3
Importance of habitat conditions (%)^3^	58	15	24	8
Niche width^4^	8.71	9.01	7.84	8.16
Germinability^5^	0.13	0.06	0.24	0.07
Competitiveness^6^	0.29	0.07	-0.08	-0.12

^1^ The value represents % seed viability after simulated endozoochory,

^2^ the values represent % seeds attached to a sheep fur after shaking 10 times,

^3^ the values represent % of variance in distribution of the species in the studied region by data on local habitat conditions and past land use at the localities.

^4^ the value represents niche width assessed for each species using a co-occurrence based approach as explained in the methods.

^5,6^ Field germinability and field competitiveness were assessed based on data from seed sowing experiment on abandoned fields in the area as described in the methods. Field germinability was assessed as the total number of individuals of particular species present in the second year following the sowing, expressed as proportion of sown viable seeds. Field competitiveness was calculated as log response ratio (LRR;) as follows LRR = ln(N_undisturbed_/N_disturbed_) where N stands for the total number of individuals established in undisturbed and disturbed plots respectively. The higher absolute value of LRR, the larger difference between seedling numbers at disturbed and undisturbed plots, positive numbers denote more seedlings being established in undisturbed plots. In this way the resulting variable express the ability of species to establish under vegetation canopy (positive LRR values), or in other words, species preference of gaps or early successional stages (negative LRR values).

### Experimental localities

**Fig 1:**
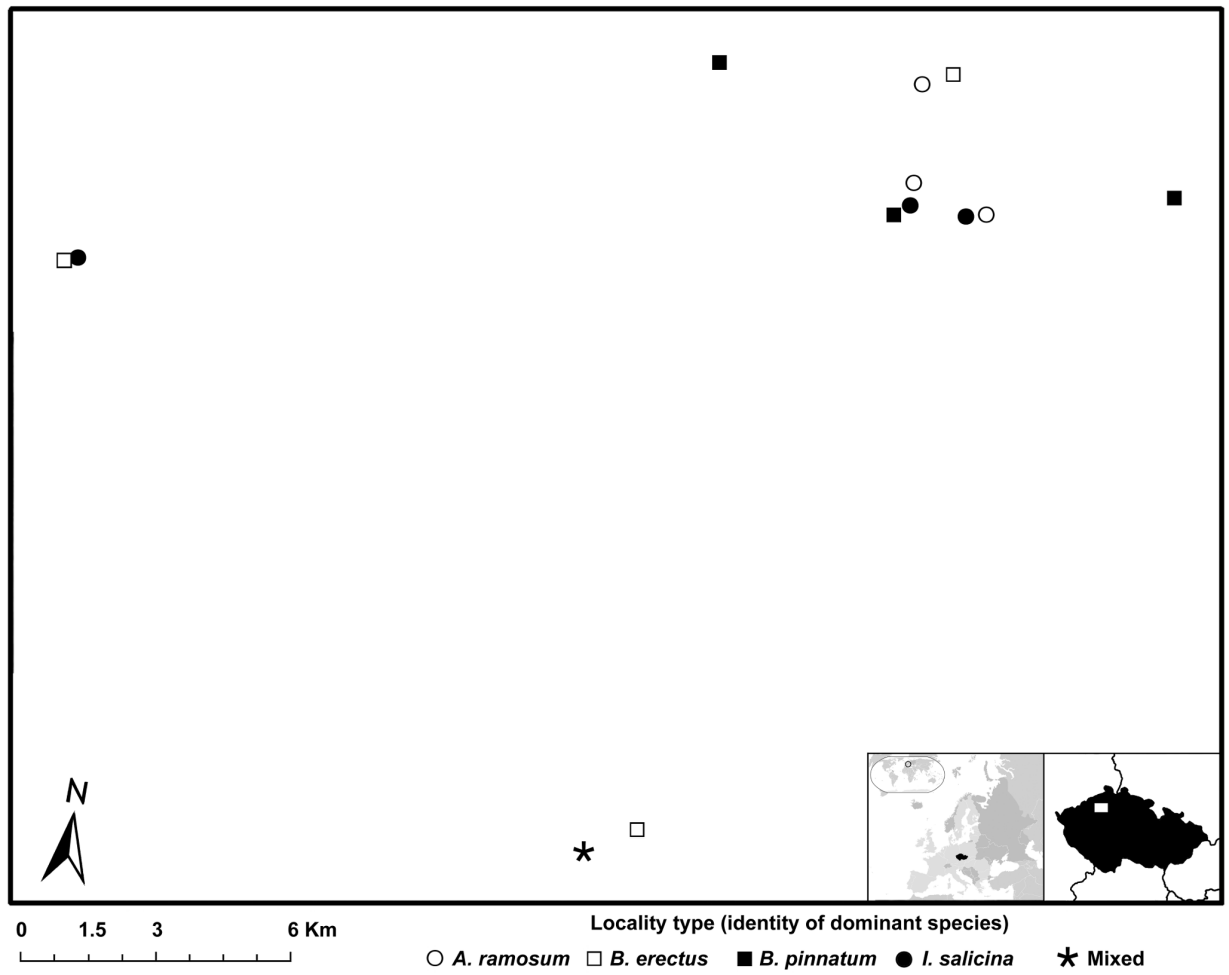
Map showing the position of the studied localities. Aerial photograph and relief model provided by the Czech Office for Surveying, Mapping and Cadastre (www.cuzk.cz).

We selected 13 localities in northern Bohemia, Czech Republic (Fig 1), for the experiment. Three localities were dominated by each of the four dominant species, i.e., 4 dominants × 3 localities, resulting in 12 localities. The remaining locality hosted all four dominant species in comparable proportions (Table 2). In the text below, we distinguish 5 locality types according to the dominant species present (*A*. *ramosum*, *B*. *pinnatum*, *B*. *erectus*, *I*. *salicina*, mixed). At all localities, we planted seedlings and sowed seeds of all species as described below. No specific permissions were required for these locations, as they were not under any type of protection at the time the study was conducted, nor were any on private land with limited access. The field studies did not involve endangered or protected species.

**Table 2:** The cover of all dominant species on each of the five locality types.

	Species							
	*A*. *ramosum*		*B*. *pinnatum*		*B*. *erectus*		*I*. *salicina*	
Locality type	Mean	SE	Mean	SE	Mean	SE	Mean	SE
*A*. *ramosum*	**39.72**	**5.48**	0.92	0.28	0	0	1.06	0.3
*B*. *pinnatum*	0*	0	**62.5**	**3.67**	0	0	0.72	0.28
*B*. *erectus*	0	0	0.19	0.12	**73.33**	**4.97**	0.17	0.07
*I*. *salicina*	0	0	0.67	0.22	0.08	0.06	**71.39**	**3.74**
Mixed	4.88	1.34	6.25	1.83	25.06	6.72	7.69	2.02

The data are % cover values based on the data collected in 1 m^2^ squares subsequently used for the sowing experiment. There were 6 squares per locality and 3 localities with a single dominant species and one mixed locality with 8 squares. The cover of the local dominant is shown in **bold**.

* indicates species presence at the locality even though it was not recorded when setting the sowing plots.

### Habitat conditions

We compared habitat conditions at the different localities to identify possible differences among them. At each locality, we collected 3 mixed soil samples, each containing a mixture of three smaller samples collected within 1 m^2^, in a dense stand of the respective dominant species. At the mixed locality, we collected four mixed samples, each in close proximity to one transplant transect (described below). We analyzed actual and potential pH using H_2_O and KCl, respectively; the content of total carbon, organic carbon, carbon in carbonates; and the total phosphorus, nitrogen, calcium, potassium and magnesium in each mixed sample [38].

To characterize local microclimatic conditions at the localities, we used TMS3 climatic stations (TOMST Co., www.tomst.com). These stations integrate three temperature sensors, humidity sensors and a datalogger. Calibrated temperature sensors (DS7505U + Maxim, Dallas Semiconductor) located in low, middle and upper positions record the temperature at 6 cm below the soil surface, on the surface and 15 cm above the surface. Humidity is measured based on the delayed passage of an electric current along a conductor at a depth between 0 and 15 cm below ground using the principle known as Time Domain Transmission. The sensor measures the delay at a frequency of 100–200 MHz. This method has the greatest resistance to salinity and temperature dependence. The inaccuracy of the measurement between the sensors under the same conditions is below ± 3% (www.tomst.com). Three stations were placed at each locality between August 2012 and August 2014, and the temperatures and moisture were recorded every 15 minutes. We used these data to calculate mean, minimum and maximum daily temperature and moisture for each station.

### Field experiment

The field translocation experiment consisted of sowing seeds and planting seedlings of all four dominants at localities with the different natural dominants. We decided to use both sowing and transplant experiments, as our previous studies in the same system showed different levels of success for these two approaches for different species (e.g., [13]). In addition, due to the very slow growth of plant species under the conditions at the studied localities (e.g., [39, 40], the seedlings did not reach the size of the transplants over the course of the experiment, and we thus evaluated different parts of the life cycle using this approach. This is also the reason we studied different plant characteristics for the sowing and planting experiments.

For the sowing experiment, we set up 6 experimental plots (1 × 1 m) at each locality. The plots were placed in a homogenous dense stand of the local dominant and were at least 2 m apart. The plots were always set up near the transects used in planting (see below). At each plot, the % cover of each of the four dominant species was recorded before the establishment of the experiment in autumn 2011 (Table 2). The plots were then disturbed, removing all of the vegetation, including roots. A central square (0.5 × 0.5 m) was divided into quarters to create subplots, and each dominant was sown into one of these subplots. The seeds were sown into the same subplots in two sequential years (autumn 2011 and 2012) because it was not possible to establish new plots in 2012 due to the small size of some localities.

The seeds for the sowing experiment were collected in 2011 and 2012 from at least 3 populations per species in the studied area and were then mixed. The seeds were stored in paper bags at room temperature before sowing. To set up the sowing experiment, we used 100 seeds per plot for *A*. *ramosum*, *B*. *pinnatum* and *B*. *erectus*. For *I*. *salicina*, which has small seeds that are difficult to count, we used a 2-ml tube full of seeds, corresponding to approximately 265 seeds, in autumn 2011. Due to low germination of *I*. *salicina* in the spring of 2012, we increased the number of sown seeds for this species to 25 ml, corresponding to approximately 3313 seeds per plot in autumn 2012. The number of seedlings in the experimental plots was recorded in June and October 2012 and in September 2013. In each recording period, the number of seedlings of each species was recorded in its sowing quarter as well as in the quarters of sowing with the other dominant species. The seedlings in the quarters sown with the other dominants were assumed to have come from the seed bank or from seed rain and served as controls.

Plants used in the seedling transplant experiment were pre-grown in an experimental garden at the Institute of Botany, Czech Academy of Sciences, Průhonice, Czech Republic. The seeds used for this purpose were collected in 2010 from at least 3 populations in the studied area. The seeds obtained from each species were mixed and used for the experiment. The seeds were sown into pots filled with garden soil in a greenhouse in January 2011. The seeds of *A*. *ramosum* were stratified in the soil in the garden for a month prior to sowing and left to germinate. In February 2011, the seedlings were individually transplanted into plug trays with a cell size of 1.5 × 1.5 cm and were kept in the greenhouse. The trays were then transferred to the experimental garden in April 2011 and were regularly watered. The seedlings were planted in the field in mid-May 2011.

At each locality, the seedlings were planted along transects. At most localities, we set up 2 transects that were approximately 24 m long (all of the transects together at each locality were always 48 m long). In the case of smaller localities, up to four shorter transects were established. The transects were always positioned in dense homogenous stands of the local dominant. In cases where a markedly different vegetation patch occurred along the transect, the transect was interrupted. At the mixed locality, we used 4 transects that were approximately 12 m long, and each of these was positioned in a vegetation patch in which one of the dominants was clearly in the minority. We planted 40 individuals of each of the 4 dominants along the transects, 30 cm apart, and alternating in the following sequence: *A*. *ramosum*, *B*. *pinnatum*, *B*. *erectus* and *I*. *salicina*. By regularly alternating the four species, we aimed to ensure that all species were planted across the whole locality. A circle with a 10 cm diameter was disturbed by removing all of the vegetation and roots up to 10 cm depth at each planting location, and the planted individual was marked. The open circles around the plants were maintained during the entire experiment by weeding at each recording time (see below). The seedlings were marked for future identification, watered immediately after planting and not further treated in any way.

The planted seedlings were revisited at the end of June/beginning of July (summer) and the end of October/beginning of November (autumn) each year (2011 to 2014), with the last recording occurring in summer 2014. During each recording period, we recorded seedling survival, length of the longest leaf for each plant (length of the longest stalk in *I*. *salicina*), number of rosettes for each plant (no. of leaves for *A*. *ramosum*) and flowering characteristics, including the number of fertile stalks and the length of the longest fertile stalk. Dead plants were replaced in summer 2011 but were not replaced afterwards.

To characterize the local effects of our model species on conspecifics and on each other, we recorded the percent cover of all four dominant species in a 30 × 30 cm square around each planted individual before the initial weeding. These data were used to determine the effect of abundance of each of the dominants on seedling size and survival.

### Feedback experiment

To test whether the performance of a dominant at a site is affected by the feedback between the plant and the soil, we set up a feedback experiment in a common garden at the Institute of Botany, Czech Academy of Sciences, Průhonice, Czech Republic. We used a classical two-phase feedback experiment (e.g. [41], [42]). To do so, we collected soil from two localities in the study region in March 2013. These localities were selected from within the vicinity of the localities used in the field experiment and represented the two most distinct soil types among the localities used in the field experiments. Both of these localities were next to dry grasslands at the edges of field roads and the soil was collected from exposed slopes along the roads. Therefore, the soil at these locations was bare and not affected by any of our dominant species or any other vegetation. We then transferred the soil to the common garden. The soil of each origin was thoroughly mixed and sieved by a sieve with a 0.5 cm mesh size to remove large stones. The experimental design described below was then applied separately to the soil of each origin.

For the first (conditioning) phase, we collected seeds of the four dominant species from the studied area between July and September 2012. Seeds of each species were collected from at least two populations and mixed. In April 2013, we sowed seeds of each dominant species into thirteen 18 × 18 × 18 cm square pots filled with soil of each origin (i.e., 4 ×13 in total) and additional 13 pots with soil of each origin served as controls. We thus had 65 (5 ×13) pots for each soil origin in total. The number of seeds sown in the conditioning phase was not counted. Instead, we sowed as many seeds as would cover the whole soil surface in one layer, approximately 5 mm apart. The seeds of *A*. *ramosum* were cold stratified in a refrigerator for 3 months before sowing. The pots were placed into a partly heated greenhouse (the temperature was prevented from dropping below 10°C). We continuously weeded out all unwanted species arriving by seed rain from all of the pots.

In October 2013, the plants, including their roots, were removed from the pots, and all of the soil that was conditioned by a single species, as well as the control soil, was thoroughly mixed, resulting in 5 different soil types for each soil origin (soil conditioned by each of the 4 dominants and a control consisting of unconditioned soil). The soil was then sifted using a sieve with a 0.5 cm mesh size to remove larger root fragments. We took 3 samples from each soil type and soil origin to analyze actual and potential pH using H_2_O and KCl, the content of total carbon, organic carbon, carbon in carbonates and the total phosphorus, nitrogen, calcium, potassium and magnesium in each sample as described above. Note that the samples for the soil analyses are not true replicates as they came from the same soil mixture. Thus, the results based on these analyses should be considered with caution. Afterwards, we mixed part of the soil conditioned by all four dominants into an even mixture, hereafter referred to as mixed soil type. As a result, we had 6 soil types in total for each soil origin.

In the second (feedback) phase of the experiment, 480 square pots of 10 × 10 × 10 cm were filled with the soil (40 pots per soil type × 6 soil types × 2 sites of soil origin). The seeds for the second phase of the experiment were collected from at least two populations between July and September 2013 and were mixed. In December 2013, we sowed 50 seeds of each dominant per pot (800 seeds for *I*. *salicina*). At the same time, seeds were also placed on Petri dishes and seed germination in the growth chamber (20°C day/5°C night with 12-hour photoperiod) was followed until all seeds either germinated or rotted (50 seeds per Petri dish (800 for *I*. *salicina*), 3 replicates per species). Each dominant was sown into 10 pots of each soil type. The pots were kept in the experimental garden to experience the winter frosts until the end of March 2014, when they were transferred to a partly heated greenhouse (the temperature was prevented from dropping below 10°C). The emerging seedlings were regularly counted. To reduce density dependence, we removed some of the seedlings to allow a maximum of 1 seedling per 6 cm^2^ (16 seedlings per pot). Most seeds germinated by the end of May 2014. At this time, all of the seedlings except for one were removed from each pot. When no seedlings survived in a pot, we transplanted into the pot a seedling of the same species, soil type and soil origin from another pot.

We measured the size of the seedlings at the end of May, in mid-July and at the beginning of September 2014. After the third measurement, the plants were harvested, separated into above- and below-ground parts, dried to a constant weight and weighed.

### Data analysis

#### Habitat conditions

To characterize habitat conditions at the experimental localities, we used a multivariate direct gradient linear analysis approach, the redundancy analysis (RDA). RDA is a form of constrained ordination which examines how much of the variation in one set of variables (the dependent variables) can be explained by other (independent) variables. It is the multivariate analog of simple linear regression [43]. The dependent variables (soil conditions) were standardized before the analyses to account for the fact that they were measured in different units. To test if the locality types (i.e., localities with different dominants) differ in soil composition, we used a Monte Carlo permutation test. In addition to comparisons of all of the locality types together, we also tested pairwise differences between different locality types. In these tests, the samples from the same locality were permuted together. The multivariate analyses were done using Canoco 4 [44].

The mean, maximum and minimum temperature and moisture data were largely correlated. After this initial exploration, we decided to include only maximum daily temperatures from the soil surface and daily minima for soil moisture, as we assumed high temperature and low moisture to be the most important limiting factors at the localities. To express the differences among the localities, we calculated the daily deviation in these values from the overall daily mean for each locality. First, we calculated the mean of each daily value across all of the dataloggers and localities and then we subtracted it from each individual value. For temperature, we expressed these deviations in degrees Celsius. For moisture, we calculated percent deviations, as the absolute values did not have a simple quantitative explanation. To test for the differences among locality types, we used analysis of variance (ANOVA) and tested for the effects of day and datalogger nested within locality, nested within locality type.

#### Field experiment

To analyze the results of the field translocation experiment, we performed tests for each target species (i.e., sown or planted species) separately. For the sowing experiment, we analyzed the number of seedlings in the plots at each recording time separately (i.e., summer 2012, autumn 2012 and summer 2013). Because the results for the single time periods were largely comparable, we decided to present only the results from the last period. The analyses were conducted using generalized linear models (GLM) with a Poisson distribution. The predictors were locality type (localities dominated by *A*. *ramosum*, *B*. *pinnatum*, *B*. *erectus*, *I*. *salicina* and the mixed locality), locality nested within locality type, plot nested within locality and cover of each dominant species in the area of the sowing plots before the experiment was established. While we also wanted to test for an interaction between cover of each dominant species and locality type, this test was not possible due to the low number of replicates (sowing plots). Such a test was, however, done for the transplant experiment below. The number of seedlings of a given sown species recorded in the subplots in which it was not sown was used as a covariate in this analysis. When plotting the data, the number of seedlings in the controls was divided by 3 (i.e., recalculated to the same area as the area of the sown subplots) and subtracted from the number of seedlings in the sown subplot.

In the field transplant experiment, we used plant size at each recording time as a dependent variable (i.e., autumn 2011, summer 2012, autumn 2012, summer 2013, autumn 2013 and summer 2014). The size of the plants at the time of planting, i.e. in summer 2011, was used as a covariate in the tests. To describe plant size, we used length of the longest leaf, number of rosettes or leaves and their product (considered to be an estimate of plant biomass, see e.g., [20]) as dependent variables. The length of the longest leaf was square root-transformed and the estimated plant biomass was log-transformed to achieve normality. We also analyzed plant survival in each recording period. The predictors of plant performance in the experiment were locality type, locality nested within locality type, cover of each dominant species and the interaction between cover of each dominant species and locality type. The effects of these predictors on leaf length and plant biomass were tested using ANOVA, the effects on the number of rosettes/leaves were tested using GLM with a Poisson distribution, and the effects on plant survival were tested using logistic regression. Because the results of the analyses based on the number of rosettes/leaves, leaf length and plant biomass were largely similar, only the results for plant biomass are presented further.

Originally, we also used the position along the transects as a covariate in the models. However, we decided to exclude this variable from the final model, as the position along the transect did not have a significant effect on plant performance. The tests were conducted for each planted dominant and for each time period separately. In addition, we performed the tests for the full dataset, as well as for the dataset excluding the mixed locality. Because the results with and without the mixed locality were largely consistent and the results among the recording periods did not strongly differ, only the results including the mixed locality and from the last recording period are presented.

*A*. *ramosum* and *I*. *salicina* never flowered in the experiment. *B*. *pinnatum* flowered only in 0.1% cases and *B*. *erectus* flowered in 1.4% cases. As a result, the data on plant flowering were not analyzed.

#### Feedback experiment

To explore the possible mechanisms explaining the intensity of plant-soil feedbacks, we tested the effects of soil origin (two source localities), soil type (soil conditioned by each target species, unconditioned soil, and the mixture of all of the conditioned soils) and their interaction on chemical composition (pH and content of nitrogen, phosphorus, carbon, calcium, magnesium and potassium) of the soil after the conditioning phase. This was done using RDA analysis using the same approaches as were used when testing for differences in soil chemistry among the locality types. In addition to analyzing the overall effect of soil type on soil composition, we also explored the pairwise differences among all pairs of soil types. Because the effect of soil type turned out to be stronger than the effect of soil origin and there was no interaction between soil origin and soil type, only the results for soil type are shown in the results.

To analyze the effects of plant-soil feedbacks on plant performance, we performed tests for each target species separately. The dependent variables were the number of germinated seedlings, plant size over the course of the experiment and aboveground and belowground biomass of the plants at the end of the experiment. The predictors were soil type, soil origin and their interaction. The effects of all predictors on plant performance were tested using GLM with a gamma distribution of the dependent variable, except for the effects on the number of germinated seedlings, which was tested using GLM with a Poisson distribution. As the results for plant size were largely similar to the results for aboveground and belowground biomass, the results for plant size are not shown or discussed further. In the results, we include tests of soil origin. In spite of few significant main effects of soil origin and their interactions, the patterns in the two soil origins were largely similar. For the purpose of the graphical presentation of the data and the comparison of the experiments described below, the data from the two soil origins were combined. Comparison of the two experiments based on data from each soil origin separately largely corresponded to the patterns based on the combined dataset.

#### Comparison of the experiments

To compare the results from the two experiments (the field experiment and the feedback experiment), the performance of each species in each soil type (i.e., at different locality types or in soils conditioned by the different species) was expressed as F_ct_ = ln(S_ct_/S_tt_), where S_ct_ is the mean performance of target species *t* grown in soil conditioned by species *c* (at the locality type dominated by species *c*) and S_tt_ is the mean performance of target species *t* grown in soil conditioned by itself (at its locality type). Performance was expressed as the number of seedlings, transplant survival and aboveground biomass for the field experiment and the number of seedlings and aboveground and belowground biomass for the feedback experiment. The value of F_ct_ thus represents the intensity of feedback of the conditioning species on the tested species (feedback experiment) or the difference in performance of the species in its own locality compared to an alien locality type (field experiment). Negative values indicate that the species performs better in soil conditioned or dominated by itself. To compare the two experiments, we calculated Pearson correlations between the F_ct_ values from both experiments based on different performance measures.

In addition to comparing plant performance, we also compared the chemical composition of the soil. To do this, we calculated the mean value of each chemical characteristic in soils from each locality type from the field experiment. In addition, we calculated the mean chemical composition of each locality type conditioned by each species in the feedback experiment. The results of these two datasets were correlated using Pearson correlations. The data from the mixed field locality and control soils from the feedback experiment were removed from these analyses.

## Results

Even though the five locality types (i.e., localities with one of the four dominants or with a mixture of all four dominants) differed in the abundance of the individual dominants (by definition), *B*. *pinnatum* and *I*. *salicina* occurred relatively frequently at all locality types (Table 2). The mixed locality hosted all of the dominants in medium abundances, i.e., lower than that at the locality dominated by the given species, but higher than at the other localities (Table 2).

### Habitat conditions

**Fig 2:**
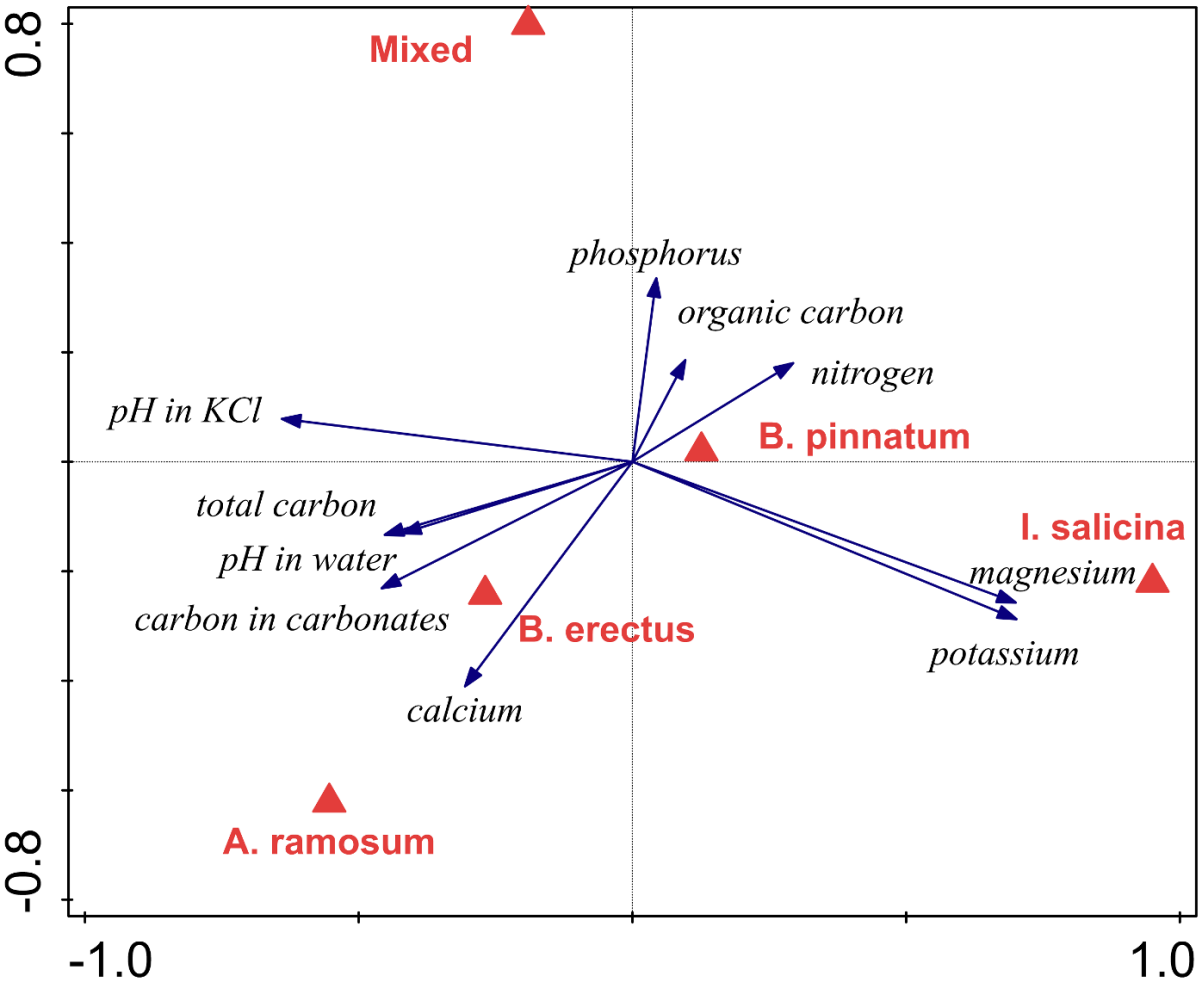
Redundancy analysis (RDA) comparing chemical composition of the soils of different locality types. The effect of locality type (identity of dominant) is marginally significant (p = 0.052, 31.7% of the total variation explained). First axis explained 21.7% and the second axis explained 5.9% of the total variation in the data.

Redundancy analysis (RDA) of the soil chemical composition indicated that the locality types were marginally significantly different in overall soil composition (p = 0.052, 31.7% of the total variance explained). The localities that deviated the most were those dominated by *I*. *salicina*, which was separated from the others along the first ordination axis (Fig 2). These localities were characterized by high magnesium and potassium content and low pH. The second ordination axis mainly separated the localities dominated by *A*. *ramosum*, which were characterized by high calcium content, from the mixed locality, which was characterized by a high phosphorus content. A pairwise comparison between locality types suggested significant differences in chemical soil composition between the localities dominated by *A*. *ramosum* and *I*. *salicina* and those dominated by *A*. *ramosum* and B. *pinnatum* (p = 0.002 in both cases). No other pairwise differences in chemical soil composition were significant (p > 0.05 in all cases).

The comparison of temperatures and moisture among the localities with different dominants showed a significant effect of locality type in all tests (p < 0.001). The localities that deviated the most were those dominated by *B*. *pinnatum*. The average daily maximum temperature was 2.2°C higher and the average soil moisture was 16% lower than that of the average locality. At the other extreme, the mixed locality was on average 4.97°C colder and 51% wetter than all of the other localities (Table 3).

**Table 3:** Deviations of maximum daily temperature at soil surface and of minimum daily soil moisture from the daily mean across all the localities.

Locality type	Temperature	Soil moisture
*A*. *ramosum*	-0.51	-6.2
*B*. *pinnatum*	*2.16*	*-16*.*0*
*B*. *erectus*	*0.30*	*-1*.*0*
*I*. *salicina*	-*0.87*	*10*.*1*
Mixed	-4.97	50.9

The data are based on values measured by three dataloggers per a locality (four dataloggers in mixed locality) from 1^st^ August 2012 to 30^th^ August 2014). All the values are significantly different from each other at p < 0.05.

### Field experiment

**Fig 3:**
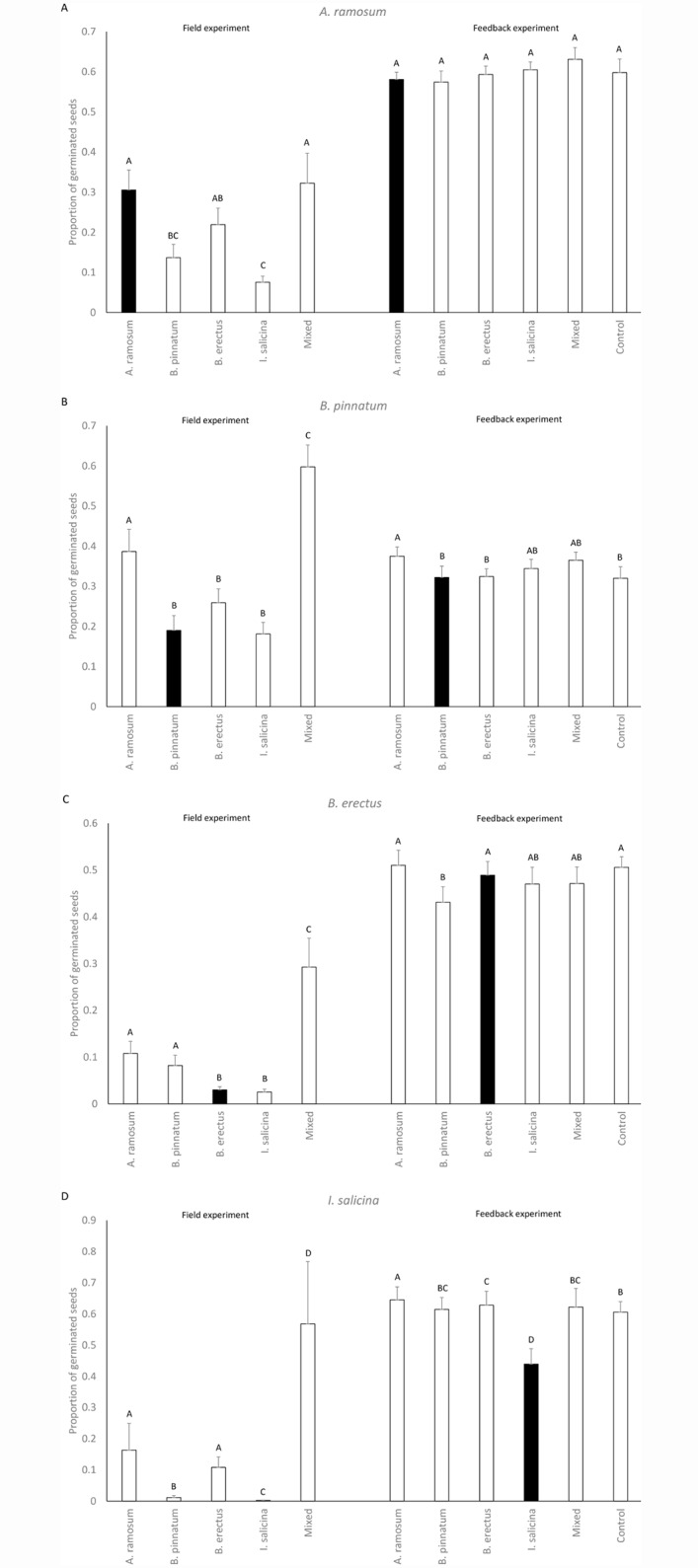
The effects of locality type (field experiment) and soil type (i.e. cultivating species in the feedback experiment) on proportion of germinated seeds of A) *Anthericum ramosum*, B) *Brachypodium pinnatum*, C) *Bromus erectus* and D) *Inula salicina*. The data from the field experiment were corrected for seed rain and germination from seedbank and multiplied by 2, to make the scale comparable between the two experiments. Columns within a panel sharing the same letter are not significantly different (p > 0.05). Black columns represent dominant species on its locality/soil type. As the patterns of the feedback in the two soil origins were largely similar, they were combined in the graphs for simplicity.

The number of seedlings in the sowing experiment was significantly affected by locality (except for *B*. *pinnatum* seedlings) as well as by locality type for all dominants (Table 4, data in S1 Table). The number of established seedlings of *A*. *ramosum* was significantly higher at its own localities, the mixed locality and localities dominated by *B*. *erectus*. In contrast, the number of seedlings of *B*. *pinnatum*, *B*. *erectus* and *I*. *salicina* at their own localities was lower than at the other localities (Fig 3). The number of seedlings of *A*. *ramosum*, *B*. *pinnatum* and *B*. *erectus* significantly decreased with their respective increasing cover at the sowing plots before removing the vegetation and setting up the sowing experiment. The number of seedlings of *I*. *salicina* increased with increasing cover of *A*. *ramosum* and *B*. *erectus* and decreased with increasing cover of *B*. *pinnatum* at the sowing plot (Table 4).

**Table 4:** The effect of locality type, locality nested within locality type, cover of each dominant species (within the area of the sowing plots before setting the experiment or in the surroundings of the transplanted individual) and its interaction with locality type on the number of seedlings, survival of transplanted plants and their aboveground biomass in field experiment.

		Target species							
		*A*. *ramosum*		*B*. *pinnatum*		*B*. *erectus*		*I*. *salicina*	
No. seedlings	Df	F	p	F	P	F	P	F	p
Loc. type	4	**16.16**	**<0.001**	**13.48**	**<0.001**	**33.04**	**<0.001**	**25.88**	**<0.001**
Locality in loc. type	8	**4.64**	**<0.001**	1.65	0.128	**3.7**	**0.001**	**7.42**	**<0.001**
*A*. *ramosum* cover	1	**3.87**	**0.054**	1.11	0.297	3.42	0.069	**45.69**	**<0.001**
*B*. *pinnatum* cover	1	0.00	0.983	**6.95**	**0.011**	0.30	0.584	**4.03**	**0.049**
*B*. *erectus* cover	1	0.03	0.854	0.36	0.553	**4.79**	**0.032**	**17.17**	**<0.001**
*I*. *salicina* cover	1	1.01	0.319	2.23	0.140	0.00	0.950	1.28	0.263
Residuals		Df = 63		Df = 63		Df = 63		Df = 63	
Survival	Df	Dev.	p	Dev.	P	Dev.	P	Dev.	p
Loc. type	4	**17.44**	**0.001**	**43.81**	**<0.001**	**18.75**	**<0.001**	**52.29**	**<0.001**
Locality in loc. type	8	**24.12**	**0.002**	**29.21**	**<0.001**	**24.13**	**0.002**	**59.05**	**<0.001**
*A*. *ramosum* cover	1	0.32	0.57	0.09	0.763	0.81	0.369	3.79	**0.052**
*B*. *pinnatum* cover	1	**6.81**	**0.009**	0.08	0.78	0.11	0.735	0.22	0.64
*B*. *erectus* cover	1	**4.25**	**0.039**	1.46	0.226	0.64	0.422	0.1	0.757
*I*. *salicina* cover	1	0.52	0.47	1.25	0.263	0	0.958	0.32	0.57
Loc. type x *B*. *erectus*	4	**6.31**	**0.043**	3.54	0.316	4.18	0.124	0.28	0.867
Residuals		Df = 343		Df = 322		DF = 375		Df = 320	
Aboveground biomass	Df	F	p	F	p	F	P	F	p
Loc. Type	**4**	**5.9**	**<0.001**	**4.87**	**<0.001**	**17.56**	**<0.001**	**12.66**	**<0.001**
Locality in loc. type	**8**	**2.82**	**0.006**	**8.78**	**<0.001**	**5.76**	**<0.001**	**4.58**	**<0.001**
*A*. *ramosum* cover	**1**	**13.21**	**<0.001**	0.04	0.841	2.81	0.094	**21.15**	**<0.001**
*B*. *pinnatum* cover	1	0.9	0.343	0.37	0.542	0.1	0.753	3.19	0.076
*B*. *erectus* cover	1	0.56	0.457	1.4	0.237	0.59	0.442	0.73	0.393
*I*. *salicina* cover	1	2.51	0.115	3.64	0.058	**8.43**	**0.004**	0.95	0.332
Loc. type x *I*. *salicina*	4	1.42	0.228	0.52	0.721	2.33	0.056	**2.58**	**0.039**
Residuals		Df = 205		Df = 224		Df = 318		Df = 160	

The interactions are shown in the table only if a given interaction was significant for at least one target species. The columns represent the sown/transplanted species. The significant values with p ≤ 0.05 are in **bold**.

Survival and biomass of all dominants in the transplant experiment were significantly affected by locality type as well as by locality (Table 4, data in S2 Table). Locality explained more variability in survival but less variability in biomass for *A*. *ramosum*, *B*. *erectus* and *I*. *salicina* than locality type. In *B*. *pinnatum*, the pattern was opposite (Table 4).

**Fig 4:**
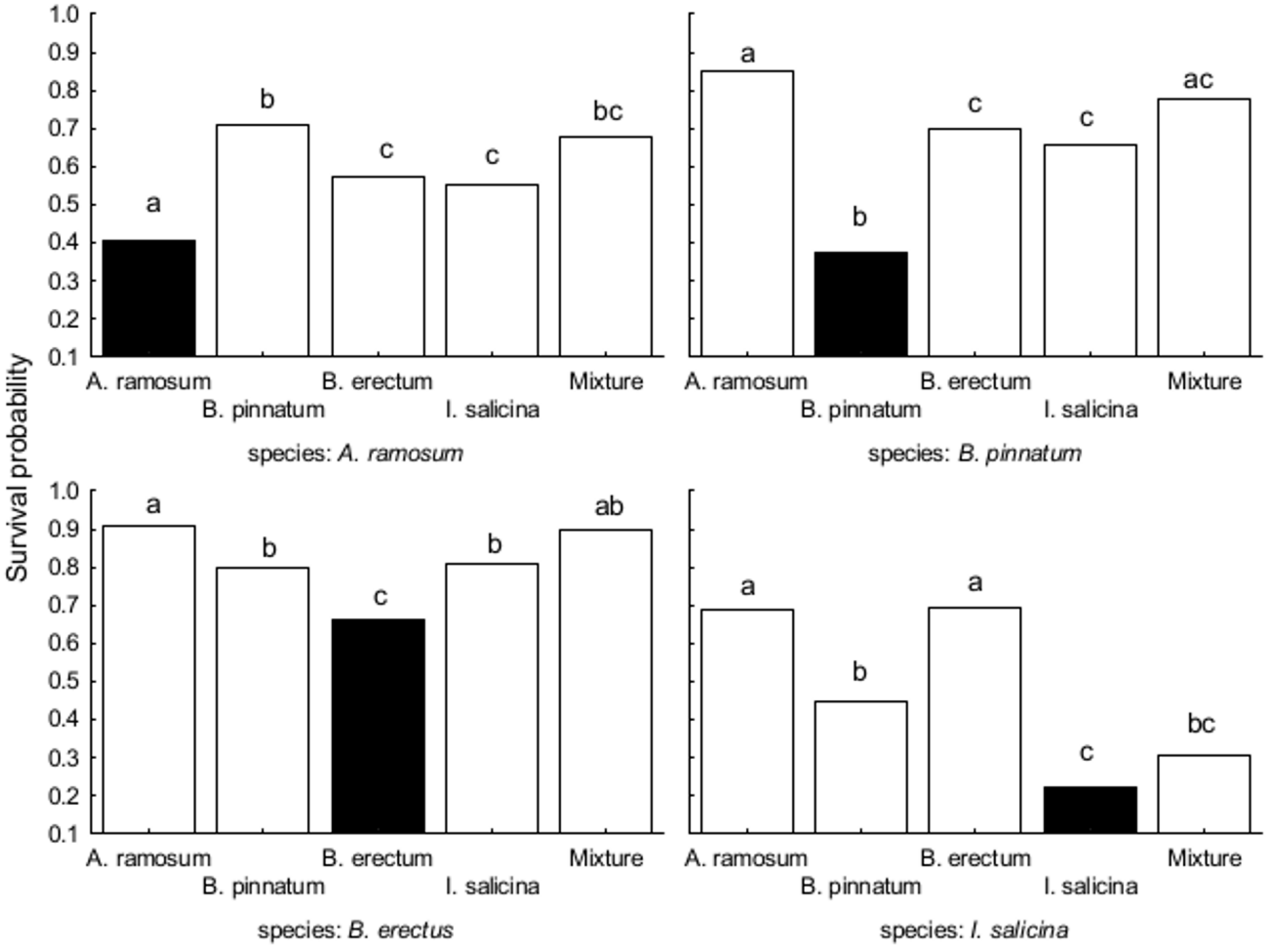
The effects of locality type on the proportion of surviving individuals in the field experiment. The effects are shown for each dominant species separately. Each panel represents one tested species, columns within the panel represent the locality types. Columns within a panel sharing the same letter are not significantly different (p > 0.05). Black columns represent dominant species on its locality type.

**Fig 5:**
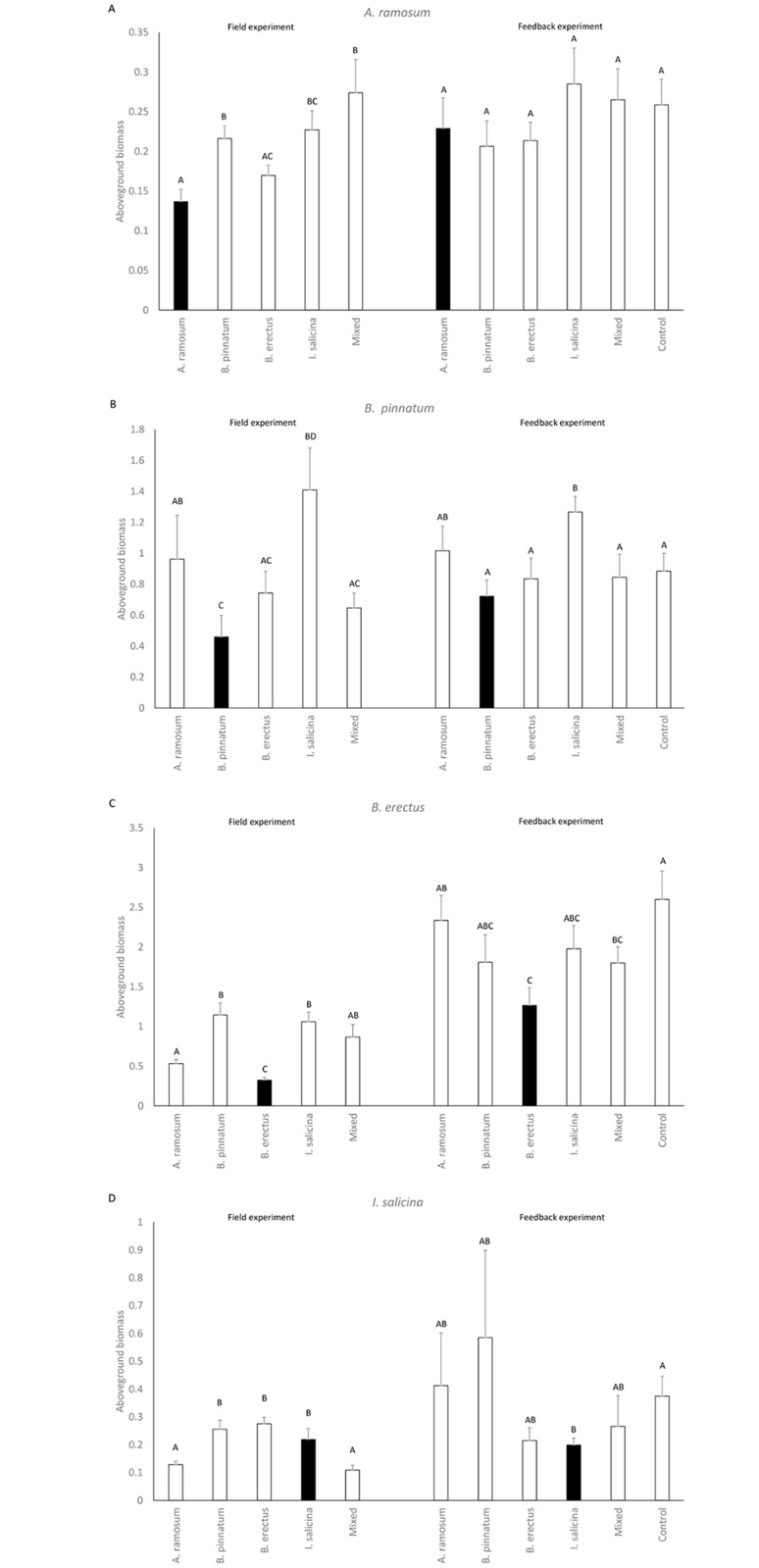
The effects of locality type (field experiment) and soil type (i.e. cultivating species in the feedback experiment) on aboveground biomass of A) *Anthericum ramosum*, B) *Brachypodium pinnatum*, C) *Bromus erectus* and D) *Inula salicina*. The data from the field experiment are estimates of biomass (number of leaves x length of the longest leaf) and were put on a scale comparable to biomass in grams from the feedback experiment. Columns within a panel sharing the same letter are not significantly different (p > 0.05). Black columns represent dominant species on its locality/soil type. As the patterns of the feedback in the two soil origins were largely similar, they were combined in the graphs for simplicity.

Survival of each species was the lowest when transplanted at the locality dominated by itself (Fig 4). In addition, *A*. *ramosum*, *B*. *pinnatum* and *B*. *erectus* growing at their own localities were smaller than at other localities. Biomass of *I*. *salicina* was similar at localities dominated by *B*. *erectus*, *B*. *pinnatum* and itself, but it was significantly lower at localities dominated by *A*. *ramosum* and at the mixed locality (Fig 5). Survival of *A*. *ramosum* decreased with the cover of *B*. *pinnatum* in its surroundings and increased with the cover of *B*. *erectus*. In addition, the effect of cover of *B*. *erectus* significantly differed between localities. Specifically, the effect was positive at the localities dominated by *B*. *erectus* and at the mixed locality, but was negative at localities dominated by *I*. *salicina*. Biomass of *A*. *ramosum* significantly increased with increasing cover of *A*. *ramosum* in its surroundings. The probability of survival of *B*. *erectus* was independent of cover of any species in its surroundings. Biomass of *B*. *erectus* significantly increased with increasing cover of *I*. *salicina* in its surroundings. Survival and biomass of *I*. *salicina* significantly increased with increasing cover of *A*. *ramosum* in its surroundings. There was also a significant interaction between locality type and cover of *I*. *salicina* for *I*. *salicina* biomass. Specifically, *I*. *salicina* biomass increased with increasing cover of *I*. *salicina* in the surroundings in its own localities but decreased at other locality types. Neither survival nor biomass of *B*. *pinnatum* was affected by the cover of any of the dominant species in its surroundings (Table 4).

### Feedback experiment

**Fig 6:**
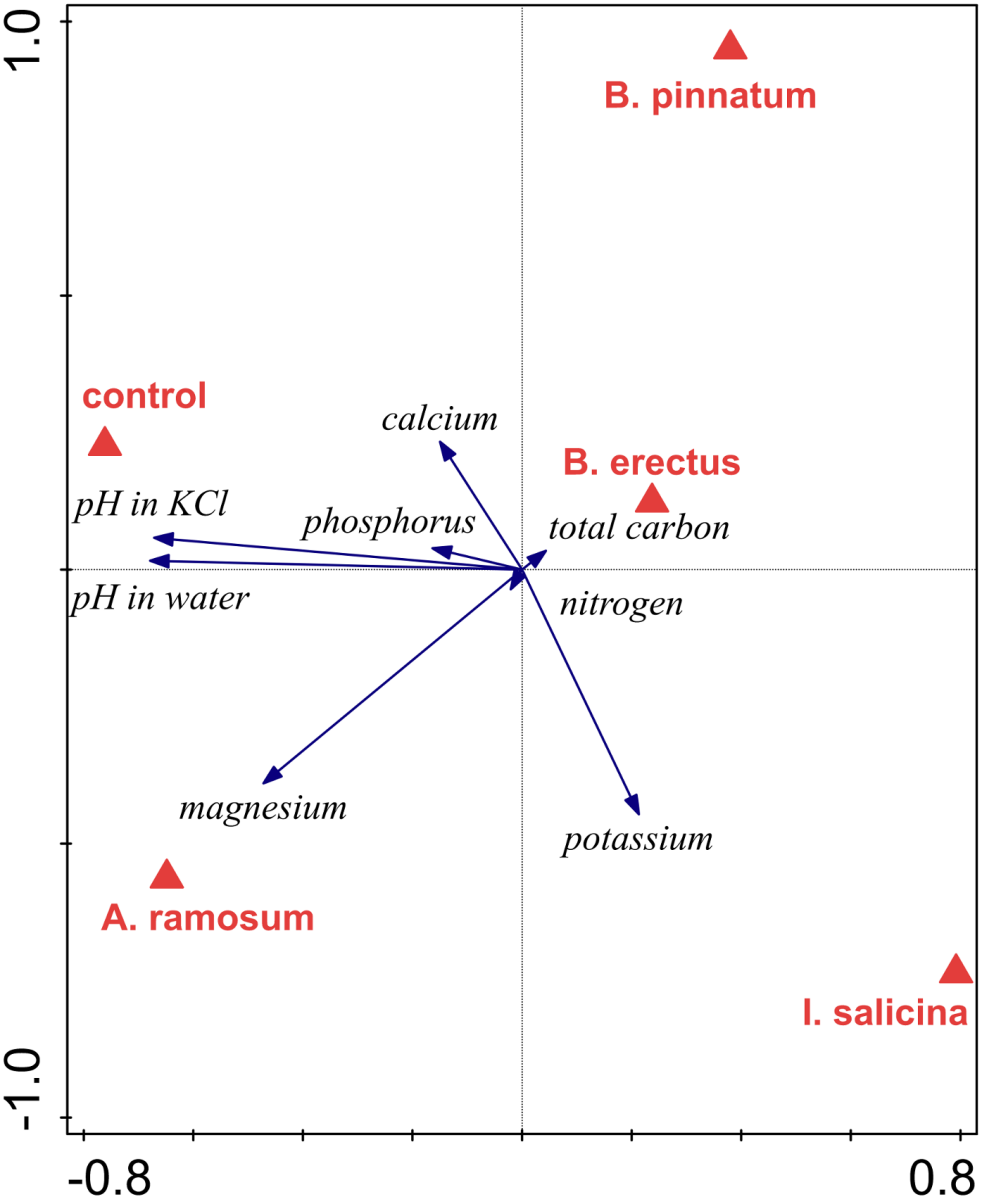
Redundancy analysis (RDA) comparing chemical composition of control soil and the soils conditioned by different dominant species in feedback experiment. The effect of conditioning is significant (p = 0.002, 23.4% of the total variance explained). First axis explained 15.4% and the second axis explained 5.2% of the total variation in the data.

Soil type had a significant effect on the chemical composition of the soil (p = 0.002, 23.4% of the total variance explained). The first ordination axis (explaining 15.4% of the total variance) clearly separated the control soil and the soil conditioned by *A*. *ramosum* from the soils conditioned by other species (Fig 6). The control soil and the soil conditioned by *A*. *ramosum* had a higher pH and contained more phosphorus, carbon and magnesium than the other soils. The second ordination axis (explaining 5.2% of the total variance) separated the soil conditioned by *B*. *pinnatum*, which had high calcium content, from the soils conditioned by *A*. *ramosum* and *I*. *salicina*, which had high magnesium and potassium content (Fig 6). All pairwise comparisons of soils conditioned by different dominants showed a significant difference (p < 0.05) except for the difference between control soil and soil conditioned by *A*. *ramosum* (p = 0.37).

In the feedback experiment, the germination of *A*. *ramosum* and *I*. *salicina*, but not other species, was significantly affected by the origin of the soil (Table 5, the data in S3 Table). In addition, seed germination in *I*. *salicina* was significantly affected by soil type and by the interaction between the soil type and the origin of the soil. *I*. *salicina* germinated worst in the soil conditioned by itself and germinated the best in the soil conditioned by *A*. *ramosum* (Table 5, Fig 3).

**Table 5:** The effect of soil origin, soil type and their interaction on the number of seedlings, and on aboveground and belowground biomass of different dominant species in feedback experiment.

		Target species							
		*A*. *ramosum*		*B*. *pinnatum*		*B*. *erectus*		*I*. *salicina*	
**Seed germ.**	Df	F	p	F	P	F	P	F	p
Conditioning	5	0.67	0.644	1.64	0.145	1.79	0.111	**168.62**	**<0.001**
Soil	**1**	**14.78**	**<0.001**	0.56	0.453	1.13	0.288	**193.74**	**<0.001**
Cond. x soil	5	1.40	0.221	0.95	0.447	2.18	0.054	**193.91**	**<0.001**
Residuals	119								
**Aboveg. biom.**	Df	F	p	F	P	F	P	F	p
Conditioning	5	0.81	0.543	2.13	0.067	**2.81**	**0.020**	**2.52**	**0.050**
Soil	1	1.45	0.231	2.60	0.110	0.02	0.901	**12.09**	**0.002**
Cond. x soil	5	1.74	0.133	**2.56**	**0.031**	**2.84**	**0.019**	0.85	0.524
Residuals	119								
**Belowg. biom.**	Df	F	p	F	P	F	P	F	p
Conditioning	5	1.41	0.227	1.90	0.100	**6.70**	**<0.001**	1.51	0.221
Soil	1	**8.39**	**0.005**	**10.72**	**0.001**	0.93	0.336	**10.08**	**0.004**
Cond. x soil	5	1.20	0.314	**3.09**	**0.012**	**4.41**	**0.001**	1.68	0.174
Residuals	119								

The significant values with p ≤ 0.05 are in **bold**.

Aboveground biomass was significantly affected by the origin of the soil in *I*. *salicina* but not the other species. In *I*. *salicina* and *B*. *erectus*, biomass was also affected by soil type. In both species, the aboveground biomass was significantly lower in soil conditioned by itself than in the control soil (Fig 5). In *B*. *pinnatum* and *B*. *erectus*, the effect of soil type interacted with the effect of soil origin (Table 5).

**Fig 7:**
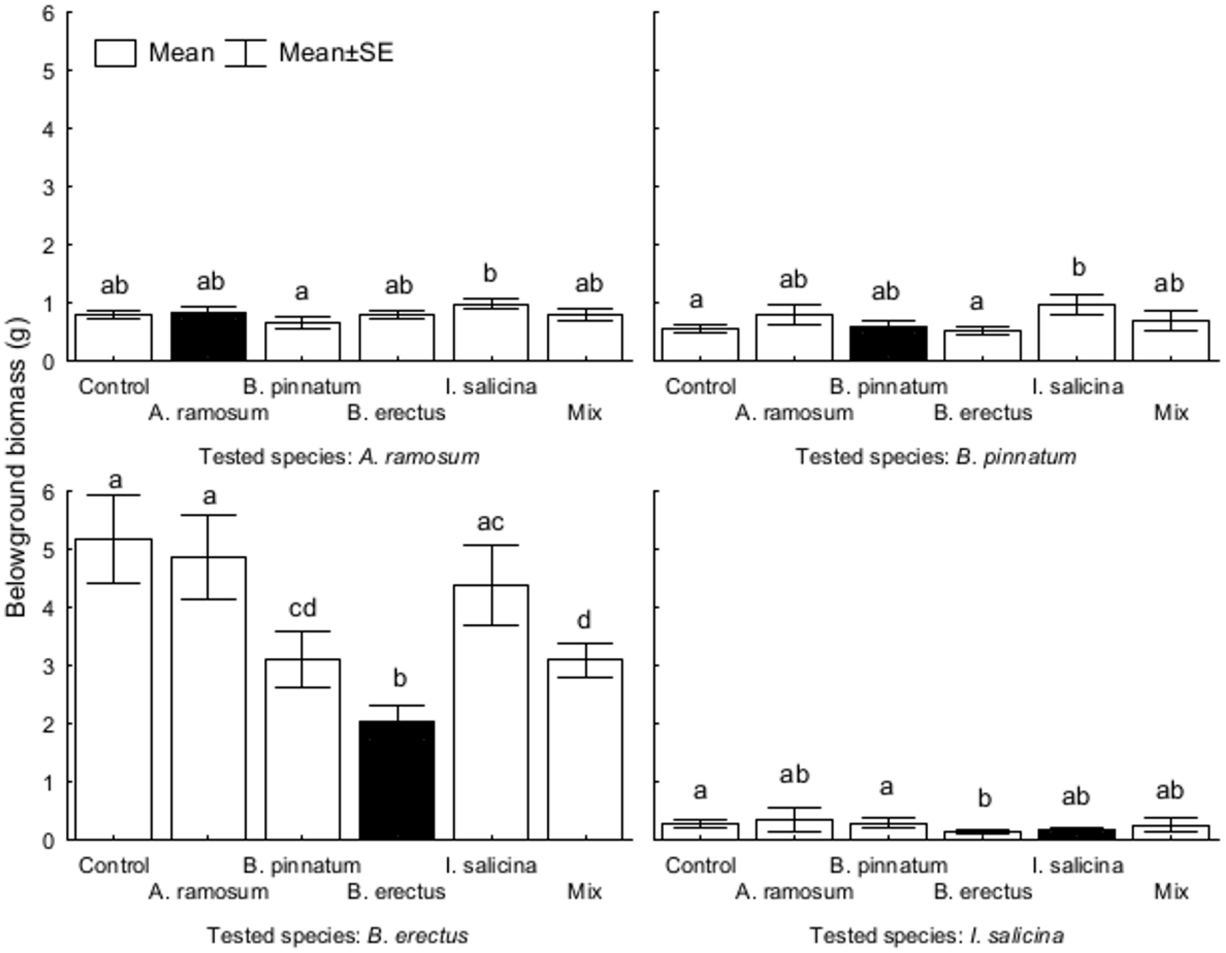
The effects of soil conditioning by each dominant species on plant belowground biomass of different dominant species in the feedback experiment. Each panel represents one tested species. The columns within each panel represent the types of soil conditioning. Columns within the panel sharing the same letter are not significantly different (p > 0.05).

Belowground biomass was significantly affected by the origin of the soil in *A*. *ramosum*, *B*. *pinnatum* and *I*. *salicina*. However, soil type had a significant effect only on the biomass of *B*. *erectus*, which was lower in soil conditioned by itself compared to the soils conditioned by the other species. There was also a significant interaction between the soil type and the origin of the soil for *B*. *pinnatum* and *B*. *erectus* (Table 5, Fig 7).

### Comparison of the field and feedback experiments

**Fig 8:**
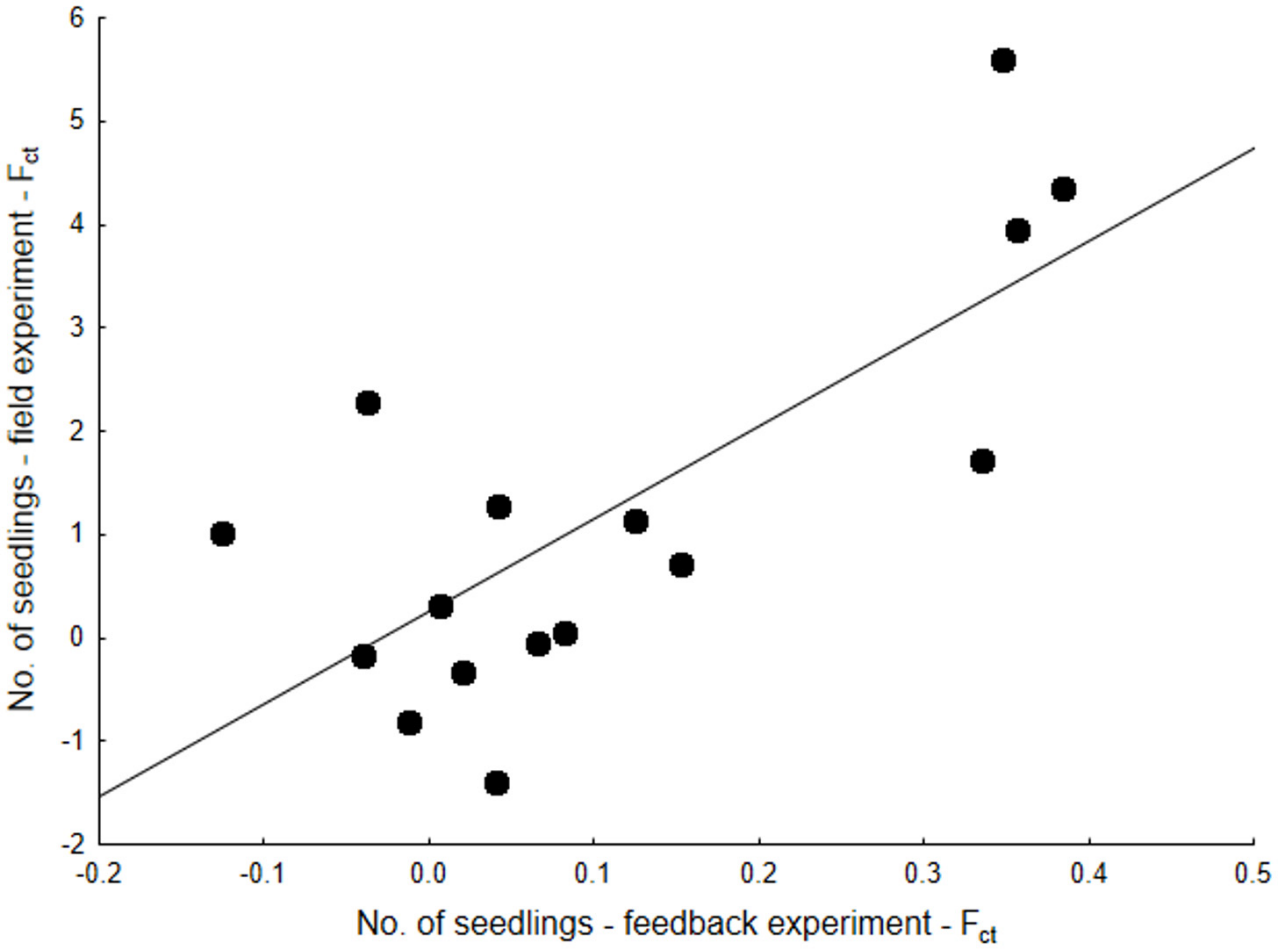
Comparison between the field and field back experiment. The graph shows the relative performances values (F_ct_) based on number of seedlings in the two experiments. As the patterns of the feedback in the two soil origins were largely similar, they were combined in the graphs for simplicity.

The number of seedlings in the feedback experiment was positively correlated with the number of seedlings (Fig 8) and plant survival and negatively correlated with aboveground biomass in the field sowing experiment (Table 6). The only other significant correlation was between aboveground and belowground biomass in the feedback experiment (Table 6). In contrast to the significant correlation in plant performance between the two experiments, we did not detect any significant correlation in soil chemistry between the experiment types (p > 0.12 in all cases). The exception was for soil pH, which was marginally significantly correlated between the two datasets (p = 0.055, r = 0.70).

**Table 6:** Pearson correlation coefficients of relative performances (F_ct_) of individuals in feedback and field experiment.

	Feedback			Field experiment		
	No. seedlings	Aboveg. biomass	Belowg. biomass	No. seedlings	Survival	Aboveg. biomass
Feedback						
No. seedlings		0.36	-0.01	**0.74**	**0.72**	-**0.79**
Aboveg. biomass	0.36		**0.76**	0.28	0.12	-0.07
Belowg. biomass	-0.01	**0.76**		0.18	-0.27	0.13
Field exp.						
No. seedlings	**0.74**	0.28	0.18		0.41	-**0.66**
Survival	**0.72**	0.12	-0.27	0.41		-0.44
Aboveg. biomass	-**0.79**	-0.07	0.13	**-0.66**	-0.44	

Significant values (p < 0.05) are in **bold**.

## Discussion

The results of the field experiment provide only limited support for the idea that local dominants perform better at the sites where they dominate. Specifically, *A*. *ramosum* germinated better at its own localities and at the mixed locality, indicating that localities where it is highly abundant are clearly more suitable for the establishment of its seedlings, even though apparently not for subsequent growth. Additionally, the number of *A*. *ramosum* seedlings was lower in plots with high cover of conspecifics before sowing. This may suggest the importance of local habitat conditions at the locality level in combination with the presence of negative local intraspecific plant-soil feedback at the plot level. In addition, localities dominated by *A*. *ramosum* significantly differed in soil composition from localities dominated by two other species (*B*. *pinnatum* and *I*. *salicina*). While this result could be interpreted as an effect of differences in habitat conditions between the localities, it could also be caused by plant-soil feedback manifested through differential nutrient acquisition by the different plant species. However, *A*. *ramosum* was the only species not having a significant effect on soil chemistry in the feedback experiment. Still, differences in nutrient acquisition could be a possible explanation, as we compared localities of *A*. *ramosum* to localities with other species that all had an effect. Finally, the results for *A*. *ramosum* are partly in line with the results of a study by Chýlová and Münzbergová [33], in which the distribution of *A*. *ramosum* was demonstrated to be the most strongly determined by environmental conditions compared to many other species in the same grassland community. In addition, Černá and Münzbergová [45] demonstrated a strong preference for its native soil in *A*. *ramosum*.

In spite of the findings described above, the overall results of this study show weak support for the environmental determination of species distributions, which is in contrast to a range of studies indicating strong habitat associations in various species (e.g., [5–10]). It is, however, in line with another body of literature showing that dispersal limitation is an important driver of species distributions and abundances in the landscape and that local habitat conditions are thus of minor importance (e.g., [13–19]).

An important discovery of our study is the fact that several of the local dominants perform significantly worse at the localities they naturally occupy than at localities dominated by the other species. In the follow-up feedback experiment, we demonstrated that the poorer performance of the species at the localities they naturally occupy could be at least partly explained by negative intraspecific plant-soil feedback ([31], [41]). Via negative intraspecific feedback, the local dominant could suppress individuals of the same species in its surroundings, e.g., by the accumulation of specialist pathogens or by a disproportional use of some nutrients that are limiting for that particular species ([32], [46]). As our sowing experiment in the field was established in disturbed plots, it is possible that the detected negative intraspecific feedback was conservative, as we removed roots of the plants and opened the space, which may have reduced some negative effects. Thus, negative intraspecific feedback in the field may be even more common than we reported.

In our feedback experiment, negative intraspecific feedbacks were clearly detected in the germination of *I*. *salicina*. A similar but weaker effect was also apparent for its aboveground biomass. In addition, *B*. *erectus* grew the poorest in its own soil, indicating that this species also suppressed itself by intraspecific plant-soil feedback. These results are largely in line with the results of the field experiment, indicating that *I*. *salicina* and *B*. *erectus* performed worse at localities dominated by their own species than at localities dominated by any other species. The performance of *I*. *salicina* and *B*. *erectus* in our field experiment could largely be explained by the intraspecific feedback effects of either biotic or abiotic factors. In contrast, there was no indication of negative intraspecific feedback in the feedback experiment for *A*. *ramosum* and *B*. *pinnatum*, but these two species still survived worse at localities dominated by themselves than at localities dominated by any other species in the field transplant experiment. Other explanations therefore need to be sought to explain this pattern.

One possible explanation could be the slow growth and low amounts of root biomass of *A*. *ramosum* and *B*. *pinnatum* in the feedback experiment. As a result, the effect of these species on the soil could be weaker than those of *I*. *salicina* and *B*. *erectus*. Still, these plants could have relatively strong negative feedback effects in the field, as the field soil is exposed to these plants for a much longer period of time and the root systems of the plants in the field are likely much larger.

Another possible explanation is that these results are caused by modifications of the biotic component of the environment that are not linked to the soil environment, as predicted by the Janzen-Connell hypothesis ([23], [24]). This could include the effects of specific pathogens or herbivores affecting aboveground plant organs, as was demonstrated in a range of studies in tropical rainforests (e.g., [47–49]). These studies suggest that possible differences in habitat requirements in different species are overwhelmed by the negative effects of accumulated pathogens and herbivores on the individuals of a particular species, i.e., a mechanism close to the plant-soil feedback, but not necessarily restricted to organisms in the soil. We are, however, not aware of any study demonstrating similar effects in temperate grassland systems. Neither are we aware of any specific pathogens or herbivores strongly affecting the aboveground parts of the plants in our system.

An alternative explanation for the lower performance of local compared to foreign dominants could be a strong effect of direct intraspecific competition, i.e., competition occurring among two individuals present at the same place at the same time, as described in classical neighborhood models of plant competition (e.g., [50]). Stronger effects of intraspecific competition in comparison with interspecific competition were previously shown in a range of studies ([51–54], but see [55]). The plots used for the field experiment were, however, disturbed to suppress direct competition. We suppose that competition was fully excluded in the sowing plots in the field experiment. In contrast, at the transplant locations in the field experiment, the disturbed plots were only 10 cm wide, and aboveground competition could therefore occur between the recording periods in spite of weeding the vegetation at each recording period. In addition, root competition could theoretically occur throughout the whole experiment in the transplant plots, as the roots extend further than the aboveground biomass and all of the dominant species are largely clonal [56]. However, we did not find any evidence that the plants would survive or grow more poorly in the case of higher conspecific density in the surroundings in the transplant experiment. The only conspecific effect in the transplant experiment was the positive effect of *A*. *ramosum* density in the surroundings on the biomass of transplanted *A*. *ramosum*. In contrast, the cover of conspecific plants in the area of the sowing experiment before the vegetation was disturbed and the sowing experiment was established, had a negative effect on the number of seedlings in three out of the four species studied. This is in line with the expectations based on negative intraspecific plant-soil feedback. While most studies on plant-soil feedback explore the effects of feedback on plant biomass, our recent study demonstrated that plant-soil feedback may be equally important in seed germination and seedling establishment [57].

Negative plant-soil feedback is commonly considered to be an important mechanism contributing to plant species coexistence, and thus increases the diversity of plant communities (e.g. [41], [58]). Several authors (e.g., [29], [59–61]) demonstrated that negative feedback is especially strong in rare species, while abundant species maintain their abundance thanks to feedbacks that are neutral or positive. In this context, the finding of possible negative feedbacks in the dominant species was not an expected result. If the dominants are truly experiencing negative plant-soil feedbacks, they should over time be replaced by other species and lose their dominant position. We do not have any direct long term data that would allow us to support or reject this expectation. However, our personal observation of the localities over the last 15 years does not indicate any strong shifts in species dominance.

The absence of any relationship between the intensity of intraspecific negative plant-soil feedback and species dominance was also demonstrated in several previous studies (e.g., [62–64]). In addition, van der Putten et al. [65] and Olff et al. [66] demonstrated that dominant species may, in fact, also experience strong intraspecific negative plant-soil feedback. However, in these systems, dominant replacement was observed as a result of the negative feedback, while no such dominant replacement is known in our system.

This controversy, i.e., stable dominance of the species in our system in spite of expected strong negative feedbacks, can have two non-exclusive explanations. First, it may be related to the fact that all four dominant species grow clonally [56] and the establishment and growth of new vegetative ramets may be much less sensitive to the effects of negative soil-feedback than the establishment of new genetic individuals. Alternatively, the plants may experience negative soil feedback, but the dynamics of the system occur very slowly and possible changes are therefore not visible over the course of a decade in these long-lived plants. This explanation is supported by the fact that previous studies from the same and in similar systems demonstrated that plants respond more strongly to past landscape structure than to the landscape structure at present ([33], [35], [67]).

From a methodological point of view, this study demonstrated that plant-soil feedback may complicate the assessment of the importance of local environmental conditions for a species. This is because negative feedback may cause the habitats dominated by the species and apparently suitable in terms of gross abiotic conditions (such as soil type, slope, moisture, and irradiation), to seem unsuitable compared to unoccupied habitats due to specific changes in soil abiotic conditions and/or soil biota. On the other hand, and for the same reason, studies assessing habitat suitability that are based only on measured habitat conditions and not using field translocation experiments may, in fact, wrongly conclude that a given locality is suitable even though the species cannot really grow there. The long-term observation of plant development in field translocation experiments or the repeated recording of species composition over a longer period of time would, however, be required to assess if reduced growth of local plants would actually lead to their replacement by other species. To further elucidate the issue, field competition experiments and field experiments using sterilized soil to eliminate the soil organisms would be helpful to test the competitive hierarchy between the species under different conditions and to identify the importance of soil biota for species performance and interactions. Finally, because our results indicate that the plant-soil feedback in our system is at least partly driven by changes in nutrient content of the soil, nutrient addition experiments should be used to test whether the negative plant-soil feedback can be caused by nutrient depletion by the dominant or if the changes in soil biota are more important.

## Supporting Information

S1 Table Primary data showing number of seedlings in the field experiment. (DOCX)

S2 Table Primary data showing plant biomass and survival in the field experiment. (DOCX)

S3 Table Primary data showing the results of the feedback experiment. (DOCX)
