# Supplementary material for: Assessment of Habitat Suitability Is Affected by Plant-Soil Feedback: Comparison of Field and Garden Experiment
Source: PLoS One. 2016 Jun 23;11(6):e0157800. doi: 10.1371/journal.pone.0157800 (PMC4919064; doi:10.1371/journal.pone.0157800)
Supplement: S1 Table — (DOCX) [file pone.0157800.s001.docx]

SX Table. Primary data showing number of seedlings in the field experiment.

| Locality | Species | Seedlings |
| --- | --- | --- |
| AR2 | AR | 5 |
| AR2 | AR | 15 |
| AR2 | AR | 29 |
| AR2 | AR | 46 |
| AR2 | AR | 41 |
| AR2 | AR | 37 |
| AR1 | AR | 38 |
| AR1 | AR | 47 |
| AR1 | AR | 50 |
| AR1 | AR | 28 |
| AR1 | AR | 40 |
| AR1 | AR | 19 |
| AR3 | AR | 20 |
| AR3 | AR | 2 |
| AR3 | AR | 28 |
| AR3 | AR | 11 |
| AR3 | AR | 23 |
| AR3 | AR | 10 |
| AR2 | BP | 20 |
| AR2 | BP | 35 |
| AR2 | BP | 29 |
| AR2 | BP | 24 |
| AR2 | BP | 45 |
| AR2 | BP | 33 |
| AR1 | BP | 24 |
| AR1 | BP | 26 |
| AR1 | BP | 20 |
| AR1 | BP | 14 |
| AR1 | BP | 15 |
| AR1 | BP | 35 |
| AR3 | BP | 43 |
| AR3 | BP | 17 |
| AR3 | BP | 17 |
| AR3 | BP | 11 |
| AR3 | BP | 29 |
| AR3 | BP | 21 |
| AR2 | BE | 5 |
| AR2 | BE | 0 |
| AR2 | BE | 2 |
| AR2 | BE | 2 |
| AR2 | BE | 14 |
| AR2 | BE | 4 |
| AR1 | BE | 9 |
| AR1 | BE | 14 |
| AR1 | BE | 20 |
| AR1 | BE | 7 |
| AR1 | BE | 9 |
| AR1 | BE | 12 |
| AR3 | BE | 4 |
| AR3 | BE | 11 |
| AR3 | BE | 3 |
| AR3 | BE | 5 |
| AR3 | BE | 2 |
| AR3 | BE | 0 |
| AR2 | IS | 0 |
| AR2 | IS | 2 |
| AR2 | IS | 0 |
| AR2 | IS | 3 |
| AR2 | IS | 24 |
| AR2 | IS | 0 |
| AR1 | IS | 0 |
| AR1 | IS | 0 |
| AR1 | IS | 0 |
| AR1 | IS | 4 |
| AR1 | IS | 0 |
| AR1 | IS | 0 |
| AR3 | IS | 171 |
| AR3 | IS | 89 |
| AR3 | IS | 140 |
| AR3 | IS | 12 |
| AR3 | IS | 36 |
| AR3 | IS | 6 |
| BE3 | AR | 5 |
| BE3 | AR | 16 |
| BE3 | AR | 9 |
| BE3 | AR | 9 |
| BE3 | AR | 1 |
| BE3 | AR | 9 |
| BE1 | AR | 12 |
| BE1 | AR | 29 |
| BE1 | AR | 0 |
| BE1 | AR | 6 |
| BE1 | AR | 12 |
| BE1 | AR | 22 |
| BE2 | AR | 36 |
| BE2 | AR | 13 |
| BE2 | AR | 25 |
| BE2 | AR | 19 |
| BE2 | AR | 6 |
| BE2 | AR | 7 |
| BE3 | BE | 3 |
| BE3 | BE | 4 |
| BE3 | BE | 0 |
| BE3 | BE | 0 |
| BE3 | BE | 2 |
| BE3 | BE | 1 |
| BE1 | BE | 2 |
| BE1 | BE | 6 |
| BE1 | BE | 3 |
| BE1 | BE | 2 |
| BE1 | BE | 2 |
| BE1 | BE | 0 |
| BE2 | BE | 6 |
| BE2 | BE | 3 |
| BE2 | BE | 4 |
| BE2 | BE | 4 |
| BE2 | BE | 1 |
| BE2 | BE | 1 |
| BE3 | BP | 13 |
| BE3 | BP | 23 |
| BE3 | BP | 13 |
| BE3 | BP | 8 |
| BE3 | BP | 12 |
| BE3 | BP | 14 |
| BE1 | BP | 21 |
| BE1 | BP | 23 |
| BE1 | BP | 3 |
| BE1 | BP | 15 |
| BE1 | BP | 14 |
| BE1 | BP | 23 |
| BE2 | BP | 31 |
| BE2 | BP | 22 |
| BE2 | BP | 38 |
| BE2 | BP | 26 |
| BE2 | BP | 18 |
| BE2 | BP | 20 |
| BE3 | IS | 2 |
| BE3 | IS | 12 |
| BE3 | IS | 53 |
| BE3 | IS | 8 |
| BE3 | IS | 56 |
| BE3 | IS | 0 |
| BE1 | IS | 0 |
| BE1 | IS | 0 |
| BE1 | IS | 0 |
| BE1 | IS | 0 |
| BE1 | IS | 0 |
| BE1 | IS | 0 |
| BE2 | IS | 58 |
| BE2 | IS | 24 |
| BE2 | IS | 36 |
| BE2 | IS | 65 |
| BE2 | IS | 61 |
| BE2 | IS | 15 |
| BP2 | AR | 10 |
| BP2 | AR | 22 |
| BP2 | AR | 12 |
| BP2 | AR | 0 |
| BP2 | AR | 16 |
| BP2 | AR | 7 |
| BP1 | AR | 8 |
| BP1 | AR | 32 |
| BP1 | AR | 21 |
| BP1 | AR | 18 |
| BP1 | AR | 17 |
| BP1 | AR | 7 |
| BP3 | AR | 2 |
| BP3 | AR | 1 |
| BP3 | AR | 2 |
| BP3 | AR | 3 |
| BP3 | AR | 7 |
| BP3 | AR | 3 |
| BP2 | BE | 1 |
| BP2 | BE | 8 |
| BP2 | BE | 2 |
| BP2 | BE | 1 |
| BP2 | BE | 1 |
| BP2 | BE | 18 |
| BP1 | BE | 10 |
| BP1 | BE | 6 |
| BP1 | BE | 14 |
| BP1 | BE | 11 |
| BP1 | BE | 8 |
| BP1 | BE | 4 |
| BP3 | BE | 0 |
| BP3 | BE | 0 |
| BP3 | BE | 3 |
| BP3 | BE | 0 |
| BP3 | BE | 3 |
| BP3 | BE | 1 |
| BP2 | BP | 1 |
| BP2 | BP | 11 |
| BP2 | BP | 14 |
| BP2 | BP | 16 |
| BP2 | BP | 16 |
| BP2 | BP | 26 |
| BP1 | BP | 31 |
| BP1 | BP | 19 |
| BP1 | BP | 7 |
| BP1 | BP | 15 |
| BP1 | BP | 20 |
| BP1 | BP | 5 |
| BP3 | BP | 12 |
| BP3 | BP | 2 |
| BP3 | BP | 16 |
| BP3 | BP | 26 |
| BP3 | BP | 33 |
| BP3 | BP | 33 |
| BP2 | IS | 0 |
| BP2 | IS | 7 |
| BP2 | IS | 1 |
| BP2 | IS | 0 |
| BP2 | IS | 0 |
| BP2 | IS | 0 |
| BP1 | IS | 0 |
| BP1 | IS | 0 |
| BP1 | IS | 0 |
| BP1 | IS | 0 |
| BP1 | IS | 0 |
| BP1 | IS | 0 |
| BP3 | IS | 3 |
| BP3 | IS | 0 |
| BP3 | IS | 1 |
| BP3 | IS | 14 |
| BP3 | IS | 4 |
| BP3 | IS | 1 |
| IS3 | AR | 9 |
| IS3 | AR | 9 |
| IS3 | AR | 1 |
| IS3 | AR | 10 |
| IS3 | AR | 15 |
| IS3 | AR | 2 |
| IS2 | AR | 1 |
| IS2 | AR | 6 |
| IS2 | AR | 0 |
| IS2 | AR | 0 |
| IS2 | AR | 0 |
| IS2 | AR | 1 |
| IS1 | AR | 10 |
| IS1 | AR | 8 |
| IS1 | AR | 3 |
| IS1 | AR | 7 |
| IS1 | AR | 14 |
| IS1 | AR | 11 |
| IS3 | BE | 1 |
| IS3 | BE | 1 |
| IS3 | BE | 1 |
| IS3 | BE | 1 |
| IS3 | BE | 0 |
| IS3 | BE | 1 |
| IS2 | BE | 7 |
| IS2 | BE | 5 |
| IS2 | BE | 0 |
| IS2 | BE | 1 |
| IS2 | BE | 2 |
| IS2 | BE | 0 |
| IS1 | BE | 2 |
| IS1 | BE | 3 |
| IS1 | BE | 5 |
| IS1 | BE | 2 |
| IS1 | BE | 4 |
| IS1 | BE | 0 |
| IS3 | BP | 16 |
| IS3 | BP | 13 |
| IS3 | BP | 12 |
| IS3 | BP | 19 |
| IS3 | BP | 21 |
| IS3 | BP | 14 |
| IS2 | BP | 13 |
| IS2 | BP | 33 |
| IS2 | BP | 1 |
| IS2 | BP | 3 |
| IS2 | BP | 1 |
| IS2 | BP | 2 |
| IS1 | BP | 12 |
| IS1 | BP | 26 |
| IS1 | BP | 14 |
| IS1 | BP | 13 |
| IS1 | BP | 21 |
| IS1 | BP | 8 |
| IS3 | IS | 1 |
| IS3 | IS | 1 |
| IS3 | IS | 0 |
| IS3 | IS | 0 |
| IS3 | IS | 2 |
| IS3 | IS | 2 |
| IS2 | IS | 0 |
| IS2 | IS | 0 |
| IS2 | IS | 1 |
| IS2 | IS | 0 |
| IS2 | IS | 0 |
| IS2 | IS | 0 |
| IS1 | IS | 3 |
| IS1 | IS | 1 |
| IS1 | IS | 4 |
| IS1 | IS | 0 |
| IS1 | IS | 2 |
| IS1 | IS | 0 |
| Mixed | AR | 15 |
| Mixed | AR | 38 |
| Mixed | AR | 33 |
| Mixed | AR | 29 |
| Mixed | AR | 12 |
| Mixed | AR | 8 |
| Mixed | AR | 11 |
| Mixed | AR | 22 |
| Mixed | BE | 31 |
| Mixed | BE | 27 |
| Mixed | BE | 15 |
| Mixed | BE | 31 |
| Mixed | BE | 14 |
| Mixed | BE | 37 |
| Mixed | BE | 20 |
| Mixed | BE | 16 |
| Mixed | BP | 50 |
| Mixed | BP | 34 |
| Mixed | BP | 48 |
| Mixed | BP | 41 |
| Mixed | BP | 45 |
| Mixed | BP | 41 |
| Mixed | BP | 47 |
| Mixed | BP | 40 |
| Mixed | IS | 153 |
| Mixed | IS | 54 |
| Mixed | IS | 82 |
| Mixed | IS | 29 |
| Mixed | IS | 73 |
| Mixed | IS | 277 |
| Mixed | IS | 12 |
| Mixed | IS | 14 |
