# Supplementary material for: Assessment of Habitat Suitability Is Affected by Plant-Soil Feedback: Comparison of Field and Garden Experiment
Source: PLoS One. 2016 Jun 23;11(6):e0157800. doi: 10.1371/journal.pone.0157800 (PMC4919064; doi:10.1371/journal.pone.0157800)
Supplement: S2 Table — (DOCX) [file pone.0157800.s002.docx]

SX Table. Primary data showing plant biomass and survival in the field experiment.

| Locality | Species | Biomass | Survival |
| --- | --- | --- | --- |
| AT1 | AT |  | 0 |
| AT1 | AT |  | 0 |
| AT1 | AT |  | 0 |
| AT1 | AT |  | 0 |
| AT1 | AT |  | 0 |
| AT1 | **AT** |  | 0 |
| AT1 | AT |  | 0 |
| AT1 | AT |  | 0 |
| AT1 | AT |  | 0 |
| AT1 | AT |  | 0 |
| AT1 | AT |  | 0 |
| AT1 | AT |  | 0 |
| AT1 | AT |  | 0 |
| AT1 | AT |  | 0 |
| AT1 | AT | 125 | 1 |
| AT1 | AT | 180 | 1 |
| AT1 | AT | 100 | 1 |
| AT1 | AT | 440 | 1 |
| AT1 | AT | 142 | 1 |
| AT1 | AT | 85 | 1 |
| AT1 | AT | 80 | 1 |
| AT1 | AT | 156 | 1 |
| AT1 | AT | 93 | 1 |
| AT1 | AT | 100 | 1 |
| AT1 | AT | 110 | 1 |
| AT1 | AT | 52 | 1 |
| AT1 | AT | 125 | 1 |
| AT1 | AT | 45 | 1 |
| AT1 | AT | 30 | 1 |
| AT1 | AT | 123 | 1 |
| AT1 | AT | 67 | 1 |
| AT1 | BRA |  | 0 |
| AT1 | BRA | 884 | 1 |
| AT1 | BRA | 2142 | 1 |
| AT1 | BRA | 592 | 1 |
| AT1 | BRA | 394 | 1 |
| AT1 | BRA | 1785 | 1 |
| AT1 | BRA | 520 | 1 |
| AT1 | BRA | 1274 | 1 |
| AT1 | BRA | 756 | 1 |
| AT1 | BRA | 1368 | 1 |
| AT1 | BRA | 3390 | 1 |
| AT1 | BRA | 375 | 1 |
| AT1 | BRA | 615 | 1 |
| AT1 | BRA | 366 | 1 |
| AT1 | BRA | 744 | 1 |
| AT1 | BRA | 170 | 1 |
| AT1 | BRA | 884 | 1 |
| AT1 | BRA | 110 | 1 |
| AT1 | BRA | 2509 | 1 |
| AT1 | BRA | 1880 | 1 |
| AT1 | BRA | 204 | 1 |
| AT1 | BRA | 540 | 1 |
| AT1 | BRA | 350 | 1 |
| AT1 | BRA | 822 | 1 |
| AT1 | BRA | 1140 | 1 |
| AT1 | BRA | 22540 | 1 |
| AT1 | BRA | 390 | 1 |
| AT1 | BRA | 1845 | 1 |
| AT1 | **BRA** | 624 | 1 |
| AT1 | BRA | 592 | 1 |
| AT1 | BRA | 514 | 1 |
| AT1 | BRA | 392 | 1 |
| AT1 | BRA | 796 | 1 |
| AT1 | BRA | 204 | 1 |
| AT1 | BRA | 1584 | 1 |
| AT1 | BRA | 440 | 1 |
| AT1 | BRA | 292 | 1 |
| AT1 | BRA | 183 | 1 |
| AT1 | BRA | 2200 | 1 |
| AT1 | BRA | 714 | 1 |
| AT1 | BRO |  | 0 |
| AT1 | BRO | 1325 | 1 |
| AT1 | BRO | 1336 | 1 |
| AT1 | BRO | 146 | 1 |
| AT1 | BRO | 498 | 1 |
| AT1 | BRO | 776 | 1 |
| AT1 | BRO | 582 | 1 |
| AT1 | BRO | 316 | 1 |
| AT1 | BRO | 1175 | 1 |
| AT1 | BRO | 228 | 1 |
| AT1 | BRO | 392 | 1 |
| AT1 | BRO | 534 | 1 |
| AT1 | BRO | 300 | 1 |
| AT1 | BRO | 206 | 1 |
| AT1 | BRO | 489 | 1 |
| AT1 | BRO | 465 | 1 |
| AT1 | BRO | 579 | 1 |
| AT1 | BRO | 981 | 1 |
| AT1 | BRO | 225 | 1 |
| AT1 | BRO | 400 | 1 |
| AT1 | BRO | 654 | 1 |
| AT1 | BRO | 892 | 1 |
| AT1 | BRO | 21 | 1 |
| AT1 | BRO | 648 | 1 |
| AT1 | BRO | 960 | 1 |
| AT1 | BRO | 283 | 1 |
| AT1 | BRO | 1160 | 1 |
| AT1 | BRO | 534 | 1 |
| AT1 | BRO | 492 | 1 |
| AT1 | BRO | 672 | 1 |
| AT1 | BRO | 592 | 1 |
| AT1 | BRO | 448 | 1 |
| AT1 | BRO | 170 | 1 |
| AT1 | BRO | 456 | 1 |
| AT1 | BRO | 476 | 1 |
| AT1 | BRO | 244 | 1 |
| AT1 | BRO | 124 | 1 |
| AT1 | BRO | 1182 | 1 |
| AT1 | BRO | 525 | 1 |
| AT1 | BRO | 1096 | 1 |
| AT1 | IN |  | 0 |
| AT1 | IN |  | 0 |
| AT1 | IN |  | 0 |
| AT1 | IN |  | 0 |
| AT1 | IN |  | 0 |
| AT1 | IN |  | 0 |
| AT1 | IN |  | 0 |
| AT1 | IN |  | 0 |
| AT1 | IN |  | 0 |
| AT1 | IN |  | 0 |
| AT1 | IN |  | 0 |
| AT1 | IN |  | 0 |
| AT1 | IN |  | 0 |
| AT1 | IN |  | 0 |
| AT1 | IN |  | 0 |
| AT1 | IN |  | 0 |
| AT1 | IN |  | 0 |
| AT1 | IN |  | 0 |
| AT1 | IN |  | 0 |
| AT1 | IN |  | 0 |
| AT1 | IN | 68 | 1 |
| AT1 | IN | 100 | 1 |
| AT1 | IN | 81 | 1 |
| AT1 | IN | 41 | 1 |
| AT1 | IN | 75 | 1 |
| AT1 | IN | 92 | 1 |
| AT1 | IN | 30 | 1 |
| AT1 | IN | 83 | 1 |
| AT1 | IN | 104 | 1 |
| AT1 | IN | 134 | 1 |
| AT1 | IN | 75 | 1 |
| AT1 | IN | 15 | 1 |
| AT1 | IN | 97 | 1 |
| AT1 | IN | 113 | 1 |
| AT1 | IN | 54 | 1 |
| AT1 | IN | 68 | 1 |
| AT1 | IN | 75 | 1 |
| AT1 | IN | 86 | 1 |
| AT1 | IN | 192 | 1 |
| AT1 | IN | 82 | 1 |
| AT2 | AT |  | 0 |
| AT2 | AT |  | 0 |
| AT2 | AT |  | 0 |
| AT2 | AT |  | 0 |
| AT2 | AT |  | 0 |
| AT2 | AT |  | 0 |
| AT2 | AT |  | 0 |
| AT2 | AT |  | 0 |
| AT2 | AT |  | 0 |
| AT2 | AT |  | 0 |
| AT2 | AT |  | 0 |
| AT2 | **AT** |  | 0 |
| AT2 | AT |  | 0 |
| AT2 | AT |  | 0 |
| AT2 | AT |  | 0 |
| AT2 | AT |  | 0 |
| AT2 | AT |  | 0 |
| AT2 | AT |  | 0 |
| AT2 | AT |  | 0 |
| AT2 | AT |  | 0 |
| AT2 | AT |  | 0 |
| AT2 | AT |  | 0 |
| AT2 | AT |  | 0 |
| AT2 | AT | 72 | 1 |
| AT2 | AT | 141 | 1 |
| AT2 | AT | 55 | 1 |
| AT2 | AT | 78 | 1 |
| AT2 | AT | 67 | 1 |
| AT2 | AT | 104 | 1 |
| AT2 | AT | 56 | 1 |
| AT2 | AT | 66 | 1 |
| AT2 | AT | 53 | 1 |
| AT2 | BRA |  | 0 |
| AT2 | BRA |  | 0 |
| AT2 | BRA |  | 0 |
| AT2 | BRA |  | 0 |
| AT2 | BRA |  | 0 |
| AT2 | BRA |  | 0 |
| AT2 | BRA |  | 0 |
| AT2 | BRA |  | 0 |
| AT2 | BRA | 3280 | 1 |
| AT2 | BRA | 1270 | 1 |
| AT2 | BRA | 562 | 1 |
| AT2 | BRA | 412 | 1 |
| AT2 | BRA | 630 | 1 |
| AT2 | BRA | 1300 | 1 |
| AT2 | BRA | 240 | 1 |
| AT2 | BRA | 166 | 1 |
| AT2 | BRA | 128 | 1 |
| AT2 | BRA | 88 | 1 |
| AT2 | BRA | 90 | 1 |
| AT2 | BRA | 393 | 1 |
| AT2 | BRA | 70 | 1 |
| AT2 | BRA | 240 | 1 |
| AT2 | BRA | 476 | 1 |
| AT2 | BRA | 992 | 1 |
| AT2 | **BRA** | 252 | 1 |
| AT2 | BRA | 201 | 1 |
| AT2 | BRA | 64 | 1 |
| AT2 | BRA | 122 | 1 |
| AT2 | BRA | 164 | 1 |
| AT2 | BRO |  | 0 |
| AT2 | BRO |  | 0 |
| AT2 | BRO |  | 0 |
| AT2 | BRO |  | 0 |
| AT2 | BRO | 301 | 1 |
| AT2 | BRO | 1002 | 1 |
| AT2 | BRO | 315 | 1 |
| AT2 | BRO | 600 | 1 |
| AT2 | BRO | 424 | 1 |
| AT2 | BRO | 520 | 1 |
| AT2 | BRO | 288 | 1 |
| AT2 | BRO | 155 | 1 |
| AT2 | BRO | 420 | 1 |
| AT2 | BRO | 261 | 1 |
| AT2 | BRO | 345 | 1 |
| AT2 | BRO | 482 | 1 |
| AT2 | BRO | 422 | 1 |
| AT2 | BRO | 225 | 1 |
| AT2 | BRO | 120 | 1 |
| AT2 | BRO | 300 | 1 |
| AT2 | BRO | 190 | 1 |
| AT2 | BRO | 465 | 1 |
| AT2 | BRO | 332 | 1 |
| AT2 | BRO | 212 | 1 |
| AT2 | BRO | 543 | 1 |
| AT2 | BRO | 480 | 1 |
| AT2 | BRO | 146 | 1 |
| AT2 | BRO | 1030 | 1 |
| AT2 | BRO | 163 | 1 |
| AT2 | BRO | 1464 | 1 |
| AT2 | BRO | 453 | 1 |
| AT2 | IN |  | 0 |
| AT2 | IN |  | 0 |
| AT2 | IN |  | 0 |
| AT2 | IN |  | 0 |
| AT2 | IN |  | 0 |
| AT2 | IN |  | 0 |
| AT2 | IN | 160 | 1 |
| AT2 | IN | 98 | 1 |
| AT2 | IN | 128 | 1 |
| AT2 | IN | 181 | 1 |
| AT2 | IN | 20 | 1 |
| AT2 | IN | 40 | 1 |
| AT2 | IN | 30 | 1 |
| AT2 | IN | 105 | 1 |
| AT2 | IN | 75 | 1 |
| AT2 | IN | 162 | 1 |
| AT2 | IN | 78 | 1 |
| AT2 | IN | 48 | 1 |
| AT2 | IN | 60 | 1 |
| AT2 | IN | 48 | 1 |
| AT2 | IN | 41 | 1 |
| AT2 | IN | 43 | 1 |
| AT2 | IN | 105 | 1 |
| AT2 | IN | 59 | 1 |
| AT2 | IN | 72 | 1 |
| AT3 | AT |  | 0 |
| AT3 | AT |  | 0 |
| AT3 | AT |  | 0 |
| AT3 | AT |  | 0 |
| AT3 | AT |  | 0 |
| AT3 | AT |  | 0 |
| AT3 | AT |  | 0 |
| AT3 | AT |  | 0 |
| AT3 | AT |  | 0 |
| AT3 | AT |  | 0 |
| AT3 | AT |  | 0 |
| AT3 | AT |  | 0 |
| AT3 | AT |  | 0 |
| AT3 | AT |  | 0 |
| AT3 | AT |  | 0 |
| AT3 | AT |  | 0 |
| AT3 | AT | 190 | 1 |
| AT3 | AT | 120 | 1 |
| AT3 | **AT** | 402 | 1 |
| AT3 | AT | 288 | 1 |
| AT3 | AT | 190 | 1 |
| AT3 | AT | 222 | 1 |
| AT3 | AT | 150 | 1 |
| AT3 | AT | 172 | 1 |
| AT3 | AT | 170 | 1 |
| AT3 | AT | 272 | 1 |
| AT3 | BRA |  | 0 |
| AT3 | BRA |  | 0 |
| AT3 | BRA |  | 0 |
| AT3 | BRA |  | 0 |
| AT3 | BRA |  | 0 |
| AT3 | BRA | 352 | 1 |
| AT3 | BRA | 690 | 1 |
| AT3 | BRA | 366 | 1 |
| AT3 | BRA | 328 | 1 |
| AT3 | BRA | 705 | 1 |
| AT3 | BRA | 384 | 1 |
| AT3 | BRA | 828 | 1 |
| AT3 | BRA | 2088 | 1 |
| AT3 | BRA | 60 | 1 |
| AT3 | BRA | 162 | 1 |
| AT3 | BRA | 272 | 1 |
| AT3 | BRA | 60 | 1 |
| AT3 | BRA | 792 | 1 |
| AT3 | BRA | 186 | 1 |
| AT3 | BRA | 378 | 1 |
| AT3 | BRA | 116 | 1 |
| AT3 | BRA | 372 | 1 |
| AT3 | BRA | 32 | 1 |
| AT3 | BRA | 240 | 1 |
| AT3 | BRA | 220 | 1 |
| AT3 | BRO |  | 0 |
| AT3 | BRO |  | 0 |
| AT3 | BRO |  | 0 |
| AT3 | BRO |  | 0 |
| AT3 | BRO |  | 0 |
| AT3 | BRO | 870 | 1 |
| AT3 | BRO | 4152 | 1 |
| AT3 | BRO | 796 | 1 |
| AT3 | BRO | 293 | 1 |
| AT3 | BRO | 752 | 1 |
| AT3 | BRO | 452 | 1 |
| AT3 | BRO | 302 | 1 |
| AT3 | BRO | 375 | 1 |
| AT3 | BRO | 1515 | 1 |
| AT3 | BRO | 652 | 1 |
| AT3 | BRO | 804 | 1 |
| AT3 | BRO | 552 | 1 |
| AT3 | BRO | 341 | 1 |
| AT3 | BRO | 193 | 1 |
| AT3 | BRO | 201 | 1 |
| AT3 | BRO | 95 | 1 |
| AT3 | BRO | 261 | 1 |
| AT3 | BRO | 92 | 1 |
| AT3 | BRO | 672 | 1 |
| AT3 | BRO | 372 | 1 |
| AT3 | BRO | 252 | 1 |
| AT3 | BRO | 182 | 1 |
| AT3 | BRO | 320 | 1 |
| AT3 | BRO | 406 | 1 |
| AT3 | BRO | 560 | 1 |
| AT3 | BRO | 340 | 1 |
| AT3 | BRO | 420 | 1 |
| AT3 | BRO | 500 | 1 |
| AT3 | BRO | 111 | 1 |
| AT3 | BRO | 700 | 1 |
| AT3 | BRO | 220 | 1 |
| AT3 | BRO | 261 | 1 |
| AT3 | IN |  | 0 |
| AT3 | IN |  | 0 |
| AT3 | IN | 255 | 1 |
| AT3 | IN | 233 | 1 |
| AT3 | IN | 476 | 1 |
| AT3 | IN | 286 | 1 |
| AT3 | IN | 430 | 1 |
| AT3 | IN | 163 | 1 |
| AT3 | IN | 160 | 1 |
| AT3 | IN | 422 | 1 |
| AT3 | IN | 200 | 1 |
| AT3 | IN | 115 | 1 |
| AT3 | IN | 50 | 1 |
| AT3 | IN | 204 | 1 |
| AT3 | IN | 67 | 1 |
| AT3 | IN | 136 | 1 |
| AT3 | IN | 168 | 1 |
| AT3 | IN | 156 | 1 |
| AT3 | IN | 264 | 1 |
| AT3 | IN | 81 | 1 |
| AT3 | IN | 138 | 1 |
| AT3 | IN | 75 | 1 |
| AT3 | IN | 178 | 1 |
| AT3 | IN | 138 | 1 |
| AT3 | IN | 342 | 1 |
| BRA1 | AT |  | 0 |
| BRA1 | AT |  | 0 |
| BRA1 | AT |  | 0 |
| BRA1 | **AT** |  | 0 |
| BRA1 | AT |  | 0 |
| BRA1 | AT |  | 0 |
| BRA1 | AT |  | 0 |
| BRA1 | AT |  | 0 |
| BRA1 | AT |  | 0 |
| BRA1 | AT |  | 0 |
| BRA1 | AT |  | 0 |
| BRA1 | AT | 86 | 1 |
| BRA1 | AT | 274 | 1 |
| BRA1 | AT | 136 | 1 |
| BRA1 | AT | 100 | 1 |
| BRA1 | AT | 115 | 1 |
| BRA1 | AT | 210 | 1 |
| BRA1 | AT | 195 | 1 |
| BRA1 | AT | 135 | 1 |
| BRA1 | AT | 136 | 1 |
| BRA1 | AT | 82 | 1 |
| BRA1 | AT | 100 | 1 |
| BRA1 | AT | 55 | 1 |
| BRA1 | AT | 90 | 1 |
| BRA1 | BRA |  | 0 |
| BRA1 | BRA |  | 0 |
| BRA1 | BRA |  | 0 |
| BRA1 | BRA |  | 0 |
| BRA1 | BRA |  | 0 |
| BRA1 | BRA |  | 0 |
| BRA1 | BRA |  | 0 |
| BRA1 | BRA |  | 0 |
| BRA1 | BRA |  | 0 |
| BRA1 | BRA |  | 0 |
| BRA1 | BRA |  | 0 |
| BRA1 | BRA |  | 0 |
| BRA1 | BRA |  | 0 |
| BRA1 | BRA |  | 0 |
| BRA1 | BRA |  | 0 |
| BRA1 | BRA |  | 0 |
| BRA1 | BRA | 37 | 1 |
| BRA1 | BRA | 185 | 1 |
| BRA1 | BRA | 88 | 1 |
| BRA1 | BRA | 54 | 1 |
| BRA1 | BRA | 191 | 1 |
| BRA1 | BRA | 1740 | 1 |
| BRA1 | BRA | 18 | 1 |
| BRA1 | BRA | 194 | 1 |
| BRA1 | BRA | 284 | 1 |
| BRA1 | BRO |  | 0 |
| BRA1 | BRO |  | 0 |
| BRA1 | BRO |  | 0 |
| BRA1 | BRO |  | 0 |
| BRA1 | BRO |  | 0 |
| BRA1 | BRO |  | 0 |
| BRA1 | BRO |  | 0 |
| BRA1 | BRO |  | 0 |
| BRA1 | BRO | 584 | 1 |
| BRA1 | BRO | 1292 | 1 |
| BRA1 | BRO | 163 | 1 |
| BRA1 | BRO | 658 | 1 |
| BRA1 | BRO | 549 | 1 |
| BRA1 | BRO | 190 | 1 |
| BRA1 | BRO | 645 | 1 |
| BRA1 | BRO | 130 | 1 |
| BRA1 | BRO | 75 | 1 |
| BRA1 | BRO | 145 | 1 |
| BRA1 | BRO | 957 | 1 |
| BRA1 | BRO | 1310 | 1 |
| BRA1 | BRO | 400 | 1 |
| BRA1 | BRO | 412 | 1 |
| BRA1 | BRO | 125 | 1 |
| BRA1 | BRO | 315 | 1 |
| BRA1 | BRO | 210 | 1 |
| BRA1 | BRO | 80 | 1 |
| BRA1 | BRO | 603 | 1 |
| BRA1 | BRO | 298 | 1 |
| BRA1 | BRO | 1410 | 1 |
| BRA1 | IN |  | 0 |
| BRA1 | IN |  | 0 |
| BRA1 | IN |  | 0 |
| BRA1 | IN |  | 0 |
| BRA1 | IN |  | 0 |
| BRA1 | IN |  | 0 |
| BRA1 | IN |  | 0 |
| BRA1 | IN |  | 0 |
| BRA1 | IN |  | 0 |
| BRA1 | IN |  | 0 |
| BRA1 | IN |  | 0 |
| BRA1 | IN |  | 0 |
| BRA1 | IN |  | 0 |
| BRA1 | IN |  | 0 |
| BRA1 | IN |  | 0 |
| BRA1 | IN |  | 0 |
| BRA1 | IN |  | 0 |
| BRA1 | IN |  | 0 |
| BRA1 | IN |  | 0 |
| BRA1 | IN |  | 0 |
| BRA1 | IN |  | 0 |
| BRA1 | IN | 160 | 1 |
| BRA1 | IN | 70 | 1 |
| BRA1 | IN | 55 | 1 |
| BRA1 | IN | 150 | 1 |
| BRA2 | AT |  | 0 |
| BRA2 | AT |  | 0 |
| BRA2 | AT |  | 0 |
| BRA2 | AT |  | 0 |
| BRA2 | AT |  | 0 |
| BRA2 | AT |  | 0 |
| BRA2 | AT | 111 | 1 |
| BRA2 | AT | 216 | 1 |
| BRA2 | AT | 166 | 1 |
| BRA2 | AT | 103 | 1 |
| BRA2 | AT | 146 | 1 |
| BRA2 | AT | 264 | 1 |
| BRA2 | **AT** | 266 | 1 |
| BRA2 | AT | 308 | 1 |
| BRA2 | AT | 382 | 1 |
| BRA2 | AT | 200 | 1 |
| BRA2 | AT | 98 | 1 |
| BRA2 | AT | 264 | 1 |
| BRA2 | AT | 154 | 1 |
| BRA2 | AT | 190 | 1 |
| BRA2 | AT | 256 | 1 |
| BRA2 | AT | 450 | 1 |
| BRA2 | AT | 262 | 1 |
| BRA2 | AT | 220 | 1 |
| BRA2 | AT | 218 | 1 |
| BRA2 | AT | 106 | 1 |
| BRA2 | AT | 252 | 1 |
| BRA2 | AT | 144 | 1 |
| BRA2 | AT | 214 | 1 |
| BRA2 | AT | 160 | 1 |
| BRA2 | AT | 238 | 1 |
| BRA2 | AT | 264 | 1 |
| BRA2 | AT | 180 | 1 |
| BRA2 | BRA |  | 0 |
| BRA2 | BRA |  | 0 |
| BRA2 | BRA |  | 0 |
| BRA2 | BRA |  | 0 |
| BRA2 | BRA |  | 0 |
| BRA2 | BRA |  | 0 |
| BRA2 | BRA |  | 0 |
| BRA2 | BRA |  | 0 |
| BRA2 | BRA |  | 0 |
| BRA2 | BRA |  | 0 |
| BRA2 | BRA |  | 0 |
| BRA2 | BRA |  | 0 |
| BRA2 | BRA |  | 0 |
| BRA2 | BRA |  | 0 |
| BRA2 | BRA |  | 0 |
| BRA2 | BRA |  | 0 |
| BRA2 | BRA |  | 0 |
| BRA2 | BRA | 179 | 1 |
| BRA2 | BRA | 180 | 1 |
| BRA2 | BRA | 170 | 1 |
| BRA2 | BRA | 335 | 1 |
| BRA2 | BRA | 160 | 1 |
| BRA2 | BRA | 3468 | 1 |
| BRA2 | **BRA** | 238 | 1 |
| BRA2 | BRA | 730 | 1 |
| BRA2 | BRO |  | 0 |
| BRA2 | BRO |  | 0 |
| BRA2 | BRO |  | 0 |
| BRA2 | BRO |  | 0 |
| BRA2 | BRO |  | 0 |
| BRA2 | BRO |  | 0 |
| BRA2 | BRO |  | 0 |
| BRA2 | BRO | 1552 | 1 |
| BRA2 | BRO | 1158 | 1 |
| BRA2 | BRO | 181 | 1 |
| BRA2 | BRO | 6020 | 1 |
| BRA2 | BRO | 792 | 1 |
| BRA2 | BRO | 666 | 1 |
| BRA2 | BRO | 2940 | 1 |
| BRA2 | BRO | 290 | 1 |
| BRA2 | BRO | 3280 | 1 |
| BRA2 | BRO | 3420 | 1 |
| BRA2 | BRO | 3950 | 1 |
| BRA2 | BRO | 4312 | 1 |
| BRA2 | BRO | 449 | 1 |
| BRA2 | BRO | 350 | 1 |
| BRA2 | BRO | 484 | 1 |
| BRA2 | BRO | 6800 | 1 |
| BRA2 | BRO | 570 | 1 |
| BRA2 | BRO | 1235 | 1 |
| BRA2 | BRO | 472 | 1 |
| BRA2 | BRO | 522 | 1 |
| BRA2 | BRO | 900 | 1 |
| BRA2 | BRO | 313 | 1 |
| BRA2 | BRO | 105 | 1 |
| BRA2 | BRO | 3168 | 1 |
| BRA2 | BRO | 1668 | 1 |
| BRA2 | IN |  | 0 |
| BRA2 | IN |  | 0 |
| BRA2 | IN |  | 0 |
| BRA2 | IN |  | 0 |
| BRA2 | IN |  | 0 |
| BRA2 | IN |  | 0 |
| BRA2 | IN |  | 0 |
| BRA2 | IN |  | 0 |
| BRA2 | IN |  | 0 |
| BRA2 | IN |  | 0 |
| BRA2 | IN |  | 0 |
| BRA2 | IN |  | 0 |
| BRA2 | IN |  | 0 |
| BRA2 | IN |  | 0 |
| BRA2 | IN | 130 | 1 |
| BRA2 | IN | 350 | 1 |
| BRA2 | IN | 146 | 1 |
| BRA2 | IN | 380 | 1 |
| BRA2 | IN | 190 | 1 |
| BRA2 | IN | 65 | 1 |
| BRA2 | IN | 201 | 1 |
| BRA2 | IN | 75 | 1 |
| BRA2 | IN | 230 | 1 |
| BRA2 | IN | 490 | 1 |
| BRA2 | IN | 286 | 1 |
| BRA2 | IN | 115 | 1 |
| BRA3 | AT |  | 0 |
| BRA3 | AT |  | 0 |
| BRA3 | AT |  | 0 |
| BRA3 | AT |  | 0 |
| BRA3 | AT |  | 0 |
| BRA3 | AT |  | 0 |
| BRA3 | AT |  | 0 |
| BRA3 | AT |  | 0 |
| BRA3 | AT |  | 0 |
| BRA3 | AT | 570 | 1 |
| BRA3 | AT | 63 | 1 |
| BRA3 | AT | 147 | 1 |
| BRA3 | AT | 86 | 1 |
| BRA3 | AT | 486 | 1 |
| BRA3 | AT | 236 | 1 |
| BRA3 | AT | 102 | 1 |
| BRA3 | AT | 152 | 1 |
| BRA3 | AT | 414 | 1 |
| BRA3 | AT | 580 | 1 |
| BRA3 | AT | 300 | 1 |
| BRA3 | AT | 280 | 1 |
| BRA3 | AT | 576 | 1 |
| BRA3 | AT | 308 | 1 |
| BRA3 | AT | 316 | 1 |
| BRA3 | AT | 220 | 1 |
| BRA3 | AT | 170 | 1 |
| BRA3 | AT | 130 | 1 |
| BRA3 | AT | 136 | 1 |
| BRA3 | AT | 180 | 1 |
| BRA3 | AT | 46 | 1 |
| BRA3 | AT | 404 | 1 |
| BRA3 | AT | 272 | 1 |
| BRA3 | AT | 111 | 1 |
| BRA3 | BRA |  | 0 |
| BRA3 | BRA |  | 0 |
| BRA3 | BRA |  | 0 |
| BRA3 | BRA |  | 0 |
| BRA3 | BRA |  | 0 |
| BRA3 | BRA |  | 0 |
| BRA3 | BRA |  | 0 |
| BRA3 | BRA |  | 0 |
| BRA3 | BRA |  | 0 |
| BRA3 | BRA |  | 0 |
| BRA3 | BRA |  | 0 |
| BRA3 | BRA |  | 0 |
| BRA3 | BRA |  | 0 |
| BRA3 | BRA |  | 0 |
| BRA3 | BRA | 759 | 1 |
| BRA3 | BRA | 264 | 1 |
| BRA3 | BRA | 1850 | 1 |
| BRA3 | BRA | 262 | 1 |
| BRA3 | BRA | 181 | 1 |
| BRA3 | BRA | 516 | 1 |
| BRA3 | BRA | 172 | 1 |
| BRA3 | BRA | 171 | 1 |
| BRA3 | BRA | 85 | 1 |
| BRA3 | BRA | 134 | 1 |
| BRA3 | BRA | 200 | 1 |
| BRA3 | BRO |  | 0 |
| BRA3 | BRO |  | 0 |
| BRA3 | BRO |  | 0 |
| BRA3 | BRO |  | 0 |
| BRA3 | BRO |  | 0 |
| BRA3 | BRO | 992 | 1 |
| BRA3 | BRO | 2580 | 1 |
| BRA3 | BRO | 470 | 1 |
| BRA3 | BRO | 1910 | 1 |
| BRA3 | BRO | 2035 | 1 |
| BRA3 | BRO | 2562 | 1 |
| BRA3 | BRO | 744 | 1 |
| BRA3 | BRO | 130 | 1 |
| BRA3 | BRO | 615 | 1 |
| BRA3 | BRO |  | 1 |
| BRA3 | BRO | 3432 | 1 |
| BRA3 | BRO | 2086 | 1 |
| BRA3 | BRO | 392 | 1 |
| BRA3 | BRO | 1695 | 1 |
| BRA3 | BRO | 534 | 1 |
| BRA3 | BRO | 193 | 1 |
| BRA3 | BRO | 272 | 1 |
| BRA3 | BRO | 144 | 1 |
| BRA3 | BRO | 620 | 1 |
| BRA3 | BRO | 424 | 1 |
| BRA3 | BRO | 1176 | 1 |
| BRA3 | BRO | 876 | 1 |
| BRA3 | BRO | 2328 | 1 |
| BRA3 | BRO | 1284 | 1 |
| BRA3 | BRO | 456 | 1 |
| BRA3 | BRO | 1992 | 1 |
| BRA3 | BRO | 211 | 1 |
| BRA3 | BRO | 801 | 1 |
| BRA3 | BRO | 121 | 1 |
| BRA3 | BRO | 206 | 1 |
| BRA3 | BRO | 669 | 1 |
| BRA3 | BRO | 147 | 1 |
| BRA3 | IN |  | 0 |
| BRA3 | IN |  | 0 |
| BRA3 | IN |  | 0 |
| BRA3 | IN |  | 0 |
| BRA3 | IN |  | 0 |
| BRA3 | IN |  | 0 |
| BRA3 | IN |  | 0 |
| BRA3 | IN |  | 0 |
| BRA3 | IN | 50 | 1 |
| BRA3 | IN | 900 | 1 |
| BRA3 | IN | 426 | 1 |
| BRA3 | IN | 138 | 1 |
| BRA3 | IN | 438 | 1 |
| BRA3 | IN | 173 | 1 |
| BRA3 | IN | 162 | 1 |
| BRA3 | IN | 98 | 1 |
| BRA3 | IN | 252 | 1 |
| BRA3 | IN | 648 | 1 |
| BRA3 | IN | 218 | 1 |
| BRA3 | IN | 382 | 1 |
| BRA3 | IN | 222 | 1 |
| BRA3 | IN | 284 | 1 |
| BRA3 | IN | 564 | 1 |
| BRA3 | IN | 486 | 1 |
| BRA3 | IN | 152 | 1 |
| BRA3 | IN | 202 | 1 |
| BRA3 | IN | 65 | 1 |
| BRO1 | AT |  | 0 |
| BRO1 | AT |  | 0 |
| BRO1 | AT |  | 0 |
| BRO1 | AT |  | 0 |
| BRO1 | **AT** |  | 0 |
| BRO1 | AT |  | 0 |
| BRO1 | AT |  | 0 |
| BRO1 | AT |  | 0 |
| BRO1 | AT |  | 0 |
| BRO1 | AT |  | 0 |
| BRO1 | AT |  | 0 |
| BRO1 | AT |  | 0 |
| BRO1 | AT |  | 0 |
| BRO1 | AT | 71 | 1 |
| BRO1 | AT | 162 | 1 |
| BRO1 | AT | 174 | 1 |
| BRO1 | AT | 148 | 1 |
| BRO1 | AT | 80 | 1 |
| BRO1 | AT | 116 | 1 |
| BRO1 | AT | 56 | 1 |
| BRO1 | AT | 312 | 1 |
| BRO1 | AT | 162 | 1 |
| BRO1 | AT | 276 | 1 |
| BRO1 | AT | 65 | 1 |
| BRO1 | AT | 237 | 1 |
| BRO1 | AT | 150 | 1 |
| BRO1 | AT | 136 | 1 |
| BRO1 | AT | 150 | 1 |
| BRO1 | AT | 243 | 1 |
| BRO1 | AT | 140 | 1 |
| BRO1 | AT | 264 | 1 |
| BRO1 | AT | 204 | 1 |
| BRO1 | AT | 168 | 1 |
| BRO1 | AT | 88 | 1 |
| BRO1 | AT | 51 | 1 |
| BRO1 | BRA |  | 0 |
| BRO1 | BRA |  | 0 |
| BRO1 | BRA |  | 0 |
| BRO1 | BRA |  | 0 |
| BRO1 | BRA |  | 0 |
| BRO1 | BRA |  | 0 |
| BRO1 | BRA |  | 0 |
| BRO1 | BRA |  | 0 |
| BRO1 | BRA |  | 0 |
| BRO1 | BRA |  | 0 |
| BRO1 | BRA |  | 0 |
| BRO1 | BRA |  | 0 |
| BRO1 | BRA |  | 0 |
| BRO1 | BRA |  | 0 |
| BRO1 | BRA | 137 | 1 |
| BRO1 | BRA | 95 | 1 |
| BRO1 | BRA | 762 | 1 |
| BRO1 | BRA | 198 | 1 |
| BRO1 | BRA | 610 | 1 |
| BRO1 | BRA | 85 | 1 |
| BRO1 | BRA | 160 | 1 |
| BRO1 | BRA | 180 | 1 |
| BRO1 | BRA | 260 | 1 |
| BRO1 | BRA | 776 | 1 |
| BRO1 | BRA | 546 | 1 |
| BRO1 | **BRA** | 72 | 1 |
| BRO1 | BRA | 98 | 1 |
| BRO1 | BRA | 99 | 1 |
| BRO1 | BRO |  | 0 |
| BRO1 | BRO |  | 0 |
| BRO1 | BRO |  | 0 |
| BRO1 | BRO |  | 0 |
| BRO1 | BRO |  | 0 |
| BRO1 | BRO |  | 0 |
| BRO1 | BRO |  | 0 |
| BRO1 | BRO | 348 | 1 |
| BRO1 | BRO | 392 | 1 |
| BRO1 | BRO | 87 | 1 |
| BRO1 | BRO | 180 | 1 |
| BRO1 | BRO | 116 | 1 |
| BRO1 | BRO | 6 | 1 |
| BRO1 | BRO | 460 | 1 |
| BRO1 | BRO | 32 | 1 |
| BRO1 | BRO | 68 | 1 |
| BRO1 | BRO | 73 | 1 |
| BRO1 | BRO | 342 | 1 |
| BRO1 | BRO | 220 | 1 |
| BRO1 | BRO | 178 | 1 |
| BRO1 | BRO | 188 | 1 |
| BRO1 | BRO | 129 | 1 |
| BRO1 | BRO | 274 | 1 |
| BRO1 | BRO | 109 | 1 |
| BRO1 | BRO | 134 | 1 |
| BRO1 | BRO | 105 | 1 |
| BRO1 | BRO | 49 | 1 |
| BRO1 | BRO | 210 | 1 |
| BRO1 | IN |  | 0 |
| BRO1 | IN |  | 0 |
| BRO1 | IN |  | 0 |
| BRO1 | IN |  | 0 |
| BRO1 | IN |  | 0 |
| BRO1 | IN |  | 0 |
| BRO1 | IN |  | 0 |
| BRO1 | IN |  | 0 |
| BRO1 | IN |  | 0 |
| BRO1 | IN |  | 0 |
| BRO1 | IN |  | 0 |
| BRO1 | IN |  | 0 |
| BRO1 | IN |  | 0 |
| BRO1 | IN |  | 0 |
| BRO1 | IN |  | 0 |
| BRO1 | IN |  | 0 |
| BRO1 | IN |  | 0 |
| BRO1 | IN | 254 | 1 |
| BRO1 | IN | 318 | 1 |
| BRO1 | IN | 98 | 1 |
| BRO1 | IN | 321 | 1 |
| BRO1 | IN | 128 | 1 |
| BRO1 | IN | 48 | 1 |
| BRO1 | IN | 74 | 1 |
| BRO1 | IN | 22 | 1 |
| BRO1 | IN | 174 | 1 |
| BRO1 | IN | 81 | 1 |
| BRO1 | IN | 6 | 1 |
| BRO2 | AT |  | 0 |
| BRO2 | AT |  | 0 |
| BRO2 | AT |  | 0 |
| BRO2 | AT |  | 0 |
| BRO2 | AT |  | 0 |
| BRO2 | AT |  | 0 |
| BRO2 | **AT** |  | 0 |
| BRO2 | AT |  | 0 |
| BRO2 | AT |  | 0 |
| BRO2 | AT |  | 0 |
| BRO2 | AT |  | 0 |
| BRO2 | AT |  | 0 |
| BRO2 | AT |  | 0 |
| BRO2 | AT |  | 0 |
| BRO2 | AT |  | 0 |
| BRO2 | AT |  | 0 |
| BRO2 | AT | 228 | 1 |
| BRO2 | AT | 130 | 1 |
| BRO2 | AT | 56 | 1 |
| BRO2 | AT | 192 | 1 |
| BRO2 | AT | 40 | 1 |
| BRO2 | AT | 246 | 1 |
| BRO2 | AT | 328 | 1 |
| BRO2 | BRA |  | 0 |
| BRO2 | BRA |  | 0 |
| BRO2 | BRA |  | 0 |
| BRO2 | BRA |  | 0 |
| BRO2 | BRA | 390 | 1 |
| BRO2 | BRA | 2330 | 1 |
| BRO2 | BRA | 5936 | 1 |
| BRO2 | BRA | 672 | 1 |
| BRO2 | BRA | 280 | 1 |
| BRO2 | BRA | 1600 | 1 |
| BRO2 | BRA | 1995 | 1 |
| BRO2 | BRA | 334 | 1 |
| BRO2 | BRA | 420 | 1 |
| BRO2 | BRA | 260 | 1 |
| BRO2 | BRA | 145 | 1 |
| BRO2 | BRA | 855 | 1 |
| BRO2 | BRA | 170 | 1 |
| BRO2 | BRA | 1107 | 1 |
| BRO2 | BRA | 801 | 1 |
| BRO2 | BRA | 552 | 1 |
| BRO2 | BRA | 1256 | 1 |
| BRO2 | **BRA** | 410 | 1 |
| BRO2 | BRA | 528 | 1 |
| BRO2 | BRA | 678 | 1 |
| BRO2 | BRA | 3900 | 1 |
| BRO2 | BRA | 255 | 1 |
| BRO2 | BRA | 306 | 1 |
| BRO2 | BRA | 850 | 1 |
| BRO2 | BRA | 332 | 1 |
| BRO2 | BRA | 382 | 1 |
| BRO2 | BRA | 856 | 1 |
| BRO2 | BRA | 3324 | 1 |
| BRO2 | BRA | 3660 | 1 |
| BRO2 | BRA | 2408 | 1 |
| BRO2 | BRO |  | 0 |
| BRO2 | BRO |  | 0 |
| BRO2 | BRO |  | 0 |
| BRO2 | BRO |  | 0 |
| BRO2 | BRO |  | 0 |
| BRO2 | BRO |  | 0 |
| BRO2 | BRO |  | 0 |
| BRO2 | BRO | 735 | 1 |
| BRO2 | BRO | 780 | 1 |
| BRO2 | BRO | 100 | 1 |
| BRO2 | BRO | 308 | 1 |
| BRO2 | BRO | 105 | 1 |
| BRO2 | BRO | 92 | 1 |
| BRO2 | BRO | 351 | 1 |
| BRO2 | BRO | 245 | 1 |
| BRO2 | BRO | 256 | 1 |
| BRO2 | BRO | 1100 | 1 |
| BRO2 | BRO | 142 | 1 |
| BRO2 | BRO | 135 | 1 |
| BRO2 | BRO | 414 | 1 |
| BRO2 | BRO | 245 | 1 |
| BRO2 | BRO | 269 | 1 |
| BRO2 | BRO | 784 | 1 |
| BRO2 | BRO | 855 | 1 |
| BRO2 | BRO | 382 | 1 |
| BRO2 | BRO | 1476 | 1 |
| BRO2 | BRO | 296 | 1 |
| BRO2 | BRO | 618 | 1 |
| BRO2 | BRO | 566 | 1 |
| BRO2 | BRO | 240 | 1 |
| BRO2 | BRO | 417 | 1 |
| BRO2 | BRO | 215 | 1 |
| BRO2 | BRO | 206 | 1 |
| BRO2 | BRO | 226 | 1 |
| BRO2 | IN |  | 0 |
| BRO2 | IN |  | 0 |
| BRO2 | IN | 241 | 1 |
| BRO2 | IN | 160 | 1 |
| BRO2 | IN | 192 | 1 |
| BRO2 | IN | 126 | 1 |
| BRO2 | IN | 435 | 1 |
| BRO2 | IN | 186 | 1 |
| BRO2 | IN | 190 | 1 |
| BRO2 | IN | 210 | 1 |
| BRO2 | IN | 145 | 1 |
| BRO2 | IN | 310 | 1 |
| BRO2 | IN | 666 | 1 |
| BRO2 | IN | 861 | 1 |
| BRO2 | IN | 520 | 1 |
| BRO2 | IN | 310 | 1 |
| BRO2 | IN | 498 | 1 |
| BRO2 | IN | 278 | 1 |
| BRO2 | IN | 798 | 1 |
| BRO2 | IN | 633 | 1 |
| BRO2 | IN | 394 | 1 |
| BRO2 | IN | 221 | 1 |
| BRO2 | IN | 678 | 1 |
| BRO2 | IN | 182 | 1 |
| BRO2 | IN | 80 | 1 |
| BRO2 | IN | 442 | 1 |
| BRO2 | IN | 202 | 1 |
| BRO2 | IN | 230 | 1 |
| BRO2 | IN | 390 | 1 |
| BRO2 | IN | 145 | 1 |
| BRO2 | IN | 167 | 1 |
| BRO2 | IN | 171 | 1 |
| BRO2 | IN | 284 | 1 |
| BRO2 | IN | 115 | 1 |
| BRO2 | IN | 482 | 1 |
| BRO2 | IN | 345 | 1 |
| BRO2 | IN | 498 | 1 |
| BRO2 | IN | 404 | 1 |
| BRO3 | AT |  | 0 |
| BRO3 | **AT** |  | 0 |
| BRO3 | AT |  | 0 |
| BRO3 | AT |  | 0 |
| BRO3 | AT |  | 0 |
| BRO3 | AT |  | 0 |
| BRO3 | AT |  | 0 |
| BRO3 | AT |  | 0 |
| BRO3 | AT |  | 0 |
| BRO3 | AT |  | 0 |
| BRO3 | AT | 252 | 1 |
| BRO3 | AT | 528 | 1 |
| BRO3 | AT | 180 | 1 |
| BRO3 | AT | 288 | 1 |
| BRO3 | AT | 284 | 1 |
| BRO3 | AT | 222 | 1 |
| BRO3 | AT | 112 | 1 |
| BRO3 | AT | 190 | 1 |
| BRO3 | AT | 90 | 1 |
| BRO3 | AT | 216 | 1 |
| BRO3 | AT | 288 | 1 |
| BRO3 | AT | 80 | 1 |
| BRO3 | AT | 130 | 1 |
| BRO3 | AT | 32 | 1 |
| BRO3 | AT | 130 | 1 |
| BRO3 | AT | 288 | 1 |
| BRO3 | AT | 126 | 1 |
| BRO3 | AT | 104 | 1 |
| BRO3 | AT | 93 | 1 |
| BRO3 | AT | 180 | 1 |
| BRO3 | AT | 57 | 1 |
| BRO3 | AT | 200 | 1 |
| BRO3 | AT | 81 | 1 |
| BRO3 | BRA |  | 0 |
| BRO3 | BRA |  | 0 |
| BRO3 | BRA |  | 0 |
| BRO3 | BRA |  | 0 |
| BRO3 | BRA |  | 0 |
| BRO3 | BRA |  | 0 |
| BRO3 | BRA |  | 0 |
| BRO3 | BRA |  | 0 |
| BRO3 | BRA | 120 | 1 |
| BRO3 | BRA | 284 | 1 |
| BRO3 | BRA | 184 | 1 |
| BRO3 | BRA | 83 | 1 |
| BRO3 | BRA | 60 | 1 |
| BRO3 | BRA | 930 | 1 |
| BRO3 | BRA | 444 | 1 |
| BRO3 | BRA | 210 | 1 |
| BRO3 | BRA | 48 | 1 |
| BRO3 | BRA | 124 | 1 |
| BRO3 | BRA | 116 | 1 |
| BRO3 | BRA | 332 | 1 |
| BRO3 | BRA | 940 | 1 |
| BRO3 | BRA | 100 | 1 |
| BRO3 | BRA | 98 | 1 |
| BRO3 | BRA | 116 | 1 |
| BRO3 | BRA | 80 | 1 |
| BRO3 | BRO |  | 0 |
| BRO3 | BRO |  | 0 |
| BRO3 | BRO |  | 0 |
| BRO3 | BRO |  | 0 |
| BRO3 | BRO |  | 0 |
| BRO3 | BRO |  | 0 |
| BRO3 | BRO |  | 0 |
| BRO3 | BRO |  | 0 |
| BRO3 | BRO |  | 0 |
| BRO3 | BRO |  | 0 |
| BRO3 | BRO |  | 0 |
| BRO3 | BRO |  | 0 |
| BRO3 | BRO |  | 0 |
| BRO3 | BRO |  | 0 |
| BRO3 | BRO |  | 0 |
| BRO3 | BRO |  | 0 |
| BRO3 | BRO |  | 0 |
| BRO3 | BRO | 256 | 1 |
| BRO3 | BRO | 226 | 1 |
| BRO3 | BRO | 181 | 1 |
| BRO3 | BRO | 274 | 1 |
| BRO3 | BRO | 172 | 1 |
| BRO3 | BRO | 300 | 1 |
| BRO3 | BRO | 402 | 1 |
| BRO3 | BRO | 264 | 1 |
| BRO3 | BRO | 25 | 1 |
| BRO3 | BRO | 228 | 1 |
| BRO3 | BRO | 903 | 1 |
| BRO3 | BRO | 705 | 1 |
| BRO3 | BRO | 573 | 1 |
| BRO3 | IN |  | 0 |
| BRO3 | IN |  | 0 |
| BRO3 | IN |  | 0 |
| BRO3 | IN |  | 0 |
| BRO3 | IN |  | 0 |
| BRO3 | IN |  | 0 |
| BRO3 | IN |  | 0 |
| BRO3 | IN |  | 0 |
| BRO3 | IN |  | 0 |
| BRO3 | IN | 252 | 1 |
| BRO3 | IN | 210 | 1 |
| BRO3 | IN | 74 | 1 |
| BRO3 | IN | 292 | 1 |
| BRO3 | IN | 272 | 1 |
| BRO3 | IN | 94 | 1 |
| BRO3 | IN | 78 | 1 |
| BRO3 | IN | 304 | 1 |
| BRO3 | IN | 290 | 1 |
| BRO3 | IN | 210 | 1 |
| BRO3 | IN | 504 | 1 |
| BRO3 | IN | 115 | 1 |
| BRO3 | IN | 266 | 1 |
| BRO3 | IN | 145 | 1 |
| BRO3 | IN | 180 | 1 |
| BRO3 | IN | 328 | 1 |
| IN1 | AT |  | 0 |
| IN1 | AT |  | 0 |
| IN1 | AT |  | 0 |
| IN1 | AT |  | 0 |
| IN1 | AT |  | 0 |
| IN1 | AT |  | 0 |
| IN1 | **AT** |  | 0 |
| IN1 | AT |  | 0 |
| IN1 | AT |  | 0 |
| IN1 | AT |  | 0 |
| IN1 | AT |  | 0 |
| IN1 | AT |  | 0 |
| IN1 | AT |  | 0 |
| IN1 | AT |  | 0 |
| IN1 | AT |  | 0 |
| IN1 | AT |  | 0 |
| IN1 | AT |  | 0 |
| IN1 | AT | 218 | 1 |
| IN1 | AT | 340 | 1 |
| IN1 | AT | 260 | 1 |
| IN1 | AT | 354 | 1 |
| IN1 | AT | 420 | 1 |
| IN1 | AT | 117 | 1 |
| IN1 | AT | 122 | 1 |
| IN1 | AT | 75 | 1 |
| IN1 | AT | 95 | 1 |
| IN1 | AT | 109 | 1 |
| IN1 | AT | 82 | 1 |
| IN1 | BRA |  | 0 |
| IN1 | BRA |  | 0 |
| IN1 | BRA |  | 0 |
| IN1 | BRA |  | 0 |
| IN1 | BRA |  | 0 |
| IN1 | BRA |  | 0 |
| IN1 | BRA | 756 | 1 |
| IN1 | BRA | 9920 | 1 |
| IN1 | BRA | 798 | 1 |
| IN1 | BRA | 508 | 1 |
| IN1 | BRA | 6810 | 1 |
| IN1 | BRA | 4338 | 1 |
| IN1 | BRA | 4774 | 1 |
| IN1 | BRA | 4872 | 1 |
| IN1 | BRA | 4392 | 1 |
| IN1 | BRA | 840 | 1 |
| IN1 | BRA | 1608 | 1 |
| IN1 | BRA | 1720 | 1 |
| IN1 | BRA | 4485 | 1 |
| IN1 | BRA | 344 | 1 |
| IN1 | BRA | 209 | 1 |
| IN1 | BRA | 4510 | 1 |
| IN1 | BRA | 2005 | 1 |
| IN1 | BRA | 684 | 1 |
| IN1 | BRA | 1107 | 1 |
| IN1 | BRA | 1705 | 1 |
| IN1 | BRA | 371 | 1 |
| IN1 | BRA | 308 | 1 |
| IN1 | BRA | 102 | 1 |
| IN1 | BRA | 756 | 1 |
| IN1 | BRA | 173 | 1 |
| IN1 | BRA | 705 | 1 |
| IN1 | BRO |  | 0 |
| IN1 | BRO |  | 0 |
| IN1 | BRO |  | 0 |
| IN1 | BRO |  | 0 |
| IN1 | BRO |  | 0 |
| IN1 | BRO |  | 0 |
| IN1 | BRO |  | 0 |
| IN1 | BRO |  | 0 |
| IN1 | BRO | 480 | 1 |
| IN1 | BRO | 316 | 1 |
| IN1 | BRO | 250 | 1 |
| IN1 | BRO | 280 | 1 |
| IN1 | BRO | 408 | 1 |
| IN1 | BRO | 4144 | 1 |
| IN1 | BRO | 466 | 1 |
| IN1 | BRO | 590 | 1 |
| IN1 | BRO | 458 | 1 |
| IN1 | BRO | 912 | 1 |
| IN1 | BRO | 586 | 1 |
| IN1 | BRO | 230 | 1 |
| IN1 | BRO | 888 | 1 |
| IN1 | BRO | 1180 | 1 |
| IN1 | BRO | 2865 | 1 |
| IN1 | BRO | 185 | 1 |
| IN1 | BRO | 192 | 1 |
| IN1 | BRO | 682 | 1 |
| IN1 | BRO | 650 | 1 |
| IN1 | BRO | 261 | 1 |
| IN1 | BRO | 170 | 1 |
| IN1 | BRO | 35 | 1 |
| IN1 | BRO | 255 | 1 |
| IN1 | BRO | 368 | 1 |
| IN1 | BRO | 450 | 1 |
| IN1 | IN |  | 0 |
| IN1 | IN |  | 0 |
| IN1 | IN |  | 0 |
| IN1 | IN |  | 0 |
| IN1 | IN |  | 0 |
| IN1 | IN |  | 0 |
| IN1 | IN |  | 0 |
| IN1 | IN |  | 0 |
| IN1 | IN |  | 0 |
| IN1 | IN |  | 0 |
| IN1 | IN |  | 0 |
| IN1 | IN |  | 0 |
| IN1 | IN |  | 0 |
| IN1 | IN |  | 0 |
| IN1 | IN |  | 0 |
| IN1 | IN |  | 0 |
| IN1 | IN |  | 0 |
| IN1 | IN |  | 0 |
| IN1 | IN |  | 0 |
| IN1 | IN |  | 0 |
| IN1 | IN |  | 0 |
| IN1 | IN |  | 0 |
| IN1 | IN |  | 0 |
| IN1 | IN |  | 0 |
| IN1 | IN | 71 | 1 |
| IN1 | IN | 189 | 1 |
| IN1 | IN | 250 | 1 |
| IN2 | AT |  | 0 |
| IN2 | AT |  | 0 |
| IN2 | AT |  | 0 |
| IN2 | AT |  | 0 |
| IN2 | AT |  | 0 |
| IN2 | AT |  | 0 |
| IN2 | AT |  | 0 |
| IN2 | **AT** |  | 0 |
| IN2 | AT |  | 0 |
| IN2 | AT |  | 0 |
| IN2 | AT |  | 0 |
| IN2 | AT |  | 0 |
| IN2 | AT | 200 | 1 |
| IN2 | AT | 163 | 1 |
| IN2 | AT | 46 | 1 |
| IN2 | AT | 212 | 1 |
| IN2 | AT | 402 | 1 |
| IN2 | AT | 232 | 1 |
| IN2 | AT | 172 | 1 |
| IN2 | AT | 544 | 1 |
| IN2 | AT | 320 | 1 |
| IN2 | AT | 970 | 1 |
| IN2 | AT | 456 | 1 |
| IN2 | AT | 456 | 1 |
| IN2 | AT | 128 | 1 |
| IN2 | AT | 336 | 1 |
| IN2 | AT | 255 | 1 |
| IN2 | AT | 504 | 1 |
| IN2 | BRA |  | 0 |
| IN2 | BRA |  | 0 |
| IN2 | BRA |  | 0 |
| IN2 | BRA |  | 0 |
| IN2 | BRA |  | 0 |
| IN2 | BRA |  | 0 |
| IN2 | BRA |  | 0 |
| IN2 | BRA |  | 0 |
| IN2 | BRA | 783 | 1 |
| IN2 | BRA | 524 | 1 |
| IN2 | BRA | 151 | 1 |
| IN2 | BRA | 71 | 1 |
| IN2 | BRA | 255 | 1 |
| IN2 | BRA | 195 | 1 |
| IN2 | BRA | 780 | 1 |
| IN2 | BRA |  | 1 |
| IN2 | BRA | 572 | 1 |
| IN2 | BRA | 2115 | 1 |
| IN2 | **BRA** | 1664 | 1 |
| IN2 | BRA | 68 | 1 |
| IN2 | BRA | 531 | 1 |
| IN2 | BRA | 680 | 1 |
| IN2 | BRA | 828 | 1 |
| IN2 | BRA | 650 | 1 |
| IN2 | BRA | 98 | 1 |
| IN2 | BRO |  | 0 |
| IN2 | BRO |  | 0 |
| IN2 | BRO |  | 0 |
| IN2 | BRO | 240 | 1 |
| IN2 | BRO | 836 | 1 |
| IN2 | BRO | 558 | 1 |
| IN2 | BRO | 92 | 1 |
| IN2 | BRO | 550 | 1 |
| IN2 | BRO | 384 | 1 |
| IN2 | BRO | 1776 | 1 |
| IN2 | BRO | 2313 | 1 |
| IN2 | BRO | 564 | 1 |
| IN2 | BRO | 684 | 1 |
| IN2 | BRO | 1104 | 1 |
| IN2 | BRO | 1113 | 1 |
| IN2 | BRO | 267 | 1 |
| IN2 | BRO | 716 | 1 |
| IN2 | BRO | 1755 | 1 |
| IN2 | BRO | 1150 | 1 |
| IN2 | BRO | 820 | 1 |
| IN2 | BRO | 2873 | 1 |
| IN2 | BRO | 3936 | 1 |
| IN2 | BRO | 1986 | 1 |
| IN2 | BRO | 1917 | 1 |
| IN2 | BRO | 1239 | 1 |
| IN2 | BRO | 900 | 1 |
| IN2 | BRO | 4690 | 1 |
| IN2 | BRO | 1140 | 1 |
| IN2 | BRO | 1002 | 1 |
| IN2 | BRO | 350 | 1 |
| IN2 | BRO | 362 | 1 |
| IN2 | BRO | 170 | 1 |
| IN2 | BRO | 900 | 1 |
| IN2 | IN |  | 0 |
| IN2 | IN |  | 0 |
| IN2 | IN |  | 0 |
| IN2 | IN |  | 0 |
| IN2 | IN |  | 0 |
| IN2 | IN |  | 0 |
| IN2 | IN |  | 0 |
| IN2 | IN |  | 0 |
| IN2 | IN |  | 0 |
| IN2 | IN |  | 0 |
| IN2 | IN |  | 0 |
| IN2 | IN |  | 0 |
| IN2 | IN |  | 0 |
| IN2 | IN |  | 0 |
| IN2 | IN |  | 0 |
| IN2 | IN |  | 0 |
| IN2 | IN |  | 0 |
| IN2 | IN |  | 0 |
| IN2 | IN |  | 0 |
| IN2 | IN | 89 | 1 |
| IN2 | IN | 236 | 1 |
| IN2 | IN | 103 | 1 |
| IN2 | IN | 489 | 1 |
| IN2 | IN | 278 | 1 |
| IN2 | IN | 67 | 1 |
| IN3 | AT |  | 0 |
| IN3 | AT |  | 0 |
| IN3 | AT |  | 0 |
| IN3 | AT |  | 0 |
| IN3 | AT |  | 0 |
| IN3 | **AT** |  | 0 |
| IN3 | AT |  | 0 |
| IN3 | AT |  | 0 |
| IN3 | AT |  | 0 |
| IN3 | AT |  | 0 |
| IN3 | AT |  | 0 |
| IN3 | AT | 103 | 1 |
| IN3 | AT | 55 | 1 |
| IN3 | AT | 56 | 1 |
| IN3 | AT | 82 | 1 |
| IN3 | AT | 33 | 1 |
| IN3 | AT | 190 | 1 |
| IN3 | AT | 190 | 1 |
| IN3 | AT | 364 | 1 |
| IN3 | AT | 106 | 1 |
| IN3 | AT | 111 | 1 |
| IN3 | AT | 82 | 1 |
| IN3 | AT | 270 | 1 |
| IN3 | AT | 83 | 1 |
| IN3 | AT | 210 | 1 |
| IN3 | AT | 144 | 1 |
| IN3 | AT | 243 | 1 |
| IN3 | AT | 236 | 1 |
| IN3 | AT | 284 | 1 |
| IN3 | AT | 192 | 1 |
| IN3 | AT | 184 | 1 |
| IN3 | AT | 138 | 1 |
| IN3 | AT | 192 | 1 |
| IN3 | BRA |  | 0 |
| IN3 | BRA |  | 0 |
| IN3 | BRA |  | 0 |
| IN3 | BRA |  | 0 |
| IN3 | BRA |  | 0 |
| IN3 | BRA |  | 0 |
| IN3 | BRA |  | 0 |
| IN3 | BRA |  | 0 |
| IN3 | BRA |  | 0 |
| IN3 | BRA |  | 0 |
| IN3 | BRA |  | 0 |
| IN3 | BRA |  | 0 |
| IN3 | BRA |  | 0 |
| IN3 | BRA |  | 0 |
| IN3 | BRA | 372 | 1 |
| IN3 | BRA | 528 | 1 |
| IN3 | BRA | 2044 | 1 |
| IN3 | BRA | 1324 | 1 |
| IN3 | BRA | 149 | 1 |
| IN3 | BRA | 70 | 1 |
| IN3 | BRA | 368 | 1 |
| IN3 | BRA | 71 | 1 |
| IN3 | BRA | 56 | 1 |
| IN3 | BRA | 920 | 1 |
| IN3 | BRA | 65 | 1 |
| IN3 | BRO |  | 0 |
| IN3 | BRO |  | 0 |
| IN3 | BRO |  | 0 |
| IN3 | BRO |  | 0 |
| IN3 | BRO |  | 0 |
| IN3 | BRO |  | 0 |
| IN3 | BRO |  | 0 |
| IN3 | BRO | 152 | 1 |
| IN3 | BRO | 410 | 1 |
| IN3 | BRO | 105 | 1 |
| IN3 | BRO | 282 | 1 |
| IN3 | BRO | 326 | 1 |
| IN3 | BRO | 664 | 1 |
| IN3 | BRO | 1836 | 1 |
| IN3 | BRO | 1866 | 1 |
| IN3 | BRO | 540 | 1 |
| IN3 | BRO | 1380 | 1 |
| IN3 | BRO | 895 | 1 |
| IN3 | BRO | 1284 | 1 |
| IN3 | BRO | 3081 | 1 |
| IN3 | BRO | 2430 | 1 |
| IN3 | BRO | 1554 | 1 |
| IN3 | BRO | 3060 | 1 |
| IN3 | BRO | 340 | 1 |
| IN3 | BRO | 1715 | 1 |
| IN3 | BRO | 1128 | 1 |
| IN3 | BRO | 2808 | 1 |
| IN3 | IN |  | 0 |
| IN3 | IN |  | 0 |
| IN3 | IN |  | 0 |
| IN3 | IN |  | 0 |
| IN3 | IN |  | 0 |
| IN3 | IN |  | 0 |
| IN3 | IN |  | 0 |
| IN3 | IN |  | 0 |
| IN3 | IN |  | 0 |
| IN3 | IN |  | 0 |
| IN3 | IN |  | 0 |
| IN3 | IN |  | 0 |
| IN3 | IN |  | 0 |
| IN3 | IN |  | 0 |
| IN3 | IN |  | 0 |
| IN3 | IN |  | 0 |
| IN3 | IN |  | 0 |
| IN3 | IN | 70 | 1 |
| IN3 | IN | 360 | 1 |
| IN3 | IN | 45 | 1 |
| IN3 | IN | 370 | 1 |
| IN3 | IN | 285 | 1 |
| IN3 | IN | 600 | 1 |
| IN3 | IN | 104 | 1 |
| IN3 | IN | 112 | 1 |
| SMES | AT |  | 0 |
| SMES | AT |  | 0 |
| SMES | AT |  | 0 |
| SMES | AT |  | 0 |
| SMES | AT |  | 0 |
| SMES | AT |  | 0 |
| SMES | AT |  | 0 |
| SMES | AT |  | 0 |
| SMES | AT |  | 0 |
| SMES | AT |  | 0 |
| SMES | AT | 282 | 1 |
| SMES | AT | 128 | 1 |
| SMES | AT | 106 | 1 |
| SMES | AT | 336 | 1 |
| SMES | AT | 256 | 1 |
| SMES | AT | 97 | 1 |
| SMES | AT | 688 | 1 |
| SMES | **AT** | 65 | 1 |
| SMES | AT | 212 | 1 |
| SMES | AT | 180 | 1 |
| SMES | AT | 140 | 1 |
| SMES | AT | 85 | 1 |
| SMES | AT | 74 | 1 |
| SMES | AT | 476 | 1 |
| SMES | AT | 603 | 1 |
| SMES | AT | 423 | 1 |
| SMES | AT | 146 | 1 |
| SMES | AT | 378 | 1 |
| SMES | AT | 453 | 1 |
| SMES | AT | 528 | 1 |
| SMES | AT | 96 | 1 |
| SMES | BRA |  | 0 |
| SMES | BRA |  | 0 |
| SMES | BRA |  | 0 |
| SMES | BRA |  | 0 |
| SMES | BRA |  | 0 |
| SMES | BRA |  | 0 |
| SMES | BRA |  | 0 |
| SMES | BRA |  | 0 |
| SMES | BRA |  | 0 |
| SMES | BRA | 1295 | 1 |
| SMES | BRA | 465 | 1 |
| SMES | BRA | 540 | 1 |
| SMES | BRA | 109 | 1 |
| SMES | BRA | 920 | 1 |
| SMES | BRA | 244 | 1 |
| SMES | BRA | 954 | 1 |
| SMES | BRA | 490 | 1 |
| SMES | BRA | 1225 | 1 |
| SMES | BRA | 1008 | 1 |
| SMES | BRA | 1288 | 1 |
| SMES | BRA | 308 | 1 |
| SMES | BRA | 304 | 1 |
| SMES | BRA | 2176 | 1 |
| SMES | BRA | 1818 | 1 |
| SMES | BRA | 1100 | 1 |
| SMES | BRA | 140 | 1 |
| SMES | BRA | 960 | 1 |
| SMES | BRA | 426 | 1 |
| SMES | BRA | 222 | 1 |
| SMES | BRA | 238 | 1 |
| SMES | **BRA** | 1184 | 1 |
| SMES | BRA | 130 | 1 |
| SMES | BRA | 392 | 1 |
| SMES | BRA | 726 | 1 |
| SMES | BRA | 120 | 1 |
| SMES | BRA | 330 | 1 |
| SMES | BRA | 122 | 1 |
| SMES | BRA | 392 | 1 |
| SMES | BRA | 245 | 1 |
| SMES | BRA | 168 | 1 |
| SMES | BRO |  | 0 |
| SMES | BRO |  | 0 |
| SMES | BRO |  | 0 |
| SMES | BRO |  | 0 |
| SMES | BRO | 1648 | 1 |
| SMES | BRO | 210 | 1 |
| SMES | BRO | 200 | 1 |
| SMES | BRO | 562 | 1 |
| SMES | BRO | 608 | 1 |
| SMES | BRO | 795 | 1 |
| SMES | BRO | 322 | 1 |
| SMES | BRO | 450 | 1 |
| SMES | BRO | 1098 | 1 |
| SMES | BRO | 554 | 1 |
| SMES | BRO | 211 | 1 |
| SMES | BRO | 209 | 1 |
| SMES | BRO | 684 | 1 |
| SMES | BRO | 990 | 1 |
| SMES | BRO | 398 | 1 |
| SMES | BRO | 324 | 1 |
| SMES | BRO | 1108 | 1 |
| SMES | BRO | 1028 | 1 |
| SMES | BRO | 336 | 1 |
| SMES | BRO | 85 | 1 |
| SMES | BRO | 764 | 1 |
| SMES | BRO | 1808 | 1 |
| SMES | BRO | 1056 | 1 |
| SMES | BRO | 110 | 1 |
| SMES | BRO | 105 | 1 |
| SMES | BRO | 450 | 1 |
| SMES | BRO | 1172 | 1 |
| SMES | BRO | 490 | 1 |
| SMES | BRO | 684 | 1 |
| SMES | BRO | 3232 | 1 |
| SMES | BRO | 484 | 1 |
| SMES | BRO | 815 | 1 |
| SMES | BRO | 1644 | 1 |
| SMES | BRO | 4992 | 1 |
| SMES | BRO | 1023 | 1 |
| SMES | BRO | 554 | 1 |
| SMES | IN |  | 0 |
| SMES | IN |  | 0 |
| SMES | IN |  | 0 |
| SMES | IN |  | 0 |
| SMES | IN |  | 0 |
| SMES | IN |  | 0 |
| SMES | IN |  | 0 |
| SMES | IN |  | 0 |
| SMES | IN |  | 0 |
| SMES | IN |  | 0 |
| SMES | IN |  | 0 |
| SMES | IN |  | 0 |
| SMES | IN |  | 0 |
| SMES | IN |  | 0 |
| SMES | IN |  | 0 |
| SMES | IN |  | 0 |
| SMES | IN |  | 0 |
| SMES | IN |  | 0 |
| SMES | IN |  | 0 |
| SMES | IN |  | 0 |
| SMES | IN |  | 0 |
| SMES | IN |  | 0 |
| SMES | IN |  | 0 |
| SMES | IN |  | 0 |
| SMES | IN |  | 0 |
| SMES | IN |  | 0 |
| SMES | IN |  | 0 |
| SMES | IN | 44 | 1 |
| SMES | IN | 69 | 1 |
| SMES | IN | 47 | 1 |
| SMES | IN | 123 | 1 |
| SMES | IN | 131 | 1 |
| SMES | IN | 42 | 1 |
| SMES | IN | 62 | 1 |
| SMES | IN | 206 | 1 |
| SMES | IN | 165 | 1 |
| SMES | IN | 198 | 1 |
| SMES | IN | 76 | 1 |
| SMES | IN | 142 | 1 |
