# Supplementary material for: Assessment of Habitat Suitability Is Affected by Plant-Soil Feedback: Comparison of Field and Garden Experiment
Source: PLoS One. 2016 Jun 23;11(6):e0157800. doi: 10.1371/journal.pone.0157800 (PMC4919064; doi:10.1371/journal.pone.0157800)
Supplement: S3 Table — (DOCX) [file pone.0157800.s003.docx]

SX Table. Primary data showing the results of the feedback experiment.

| conditioningT | soil | Tested species | Seedlings | Roots | Shoots |
| --- | --- | --- | --- | --- | --- |
| A. ramosum | A | A. ramosum | 28 | 0.604 | 0.115 |
| A. ramosum | A | A. ramosum | 30 | 1.357 | 0.209 |
| A. ramosum | A | A. ramosum | 28 | 1.19 | 0.215 |
| A. ramosum | A | A. ramosum | 33 | 0.93 | 0.224 |
| A. ramosum | A | A. ramosum | 34 | 1.168 | 0.191 |
| A. ramosum | A | A. ramosum | 26 | 1.514 | 0.642 |
| A. ramosum | A | A. ramosum | 31 | 0.717 | 0.187 |
| A. ramosum | A | A. ramosum | 28 | 0.774 | 0.341 |
| A. ramosum | A | A. ramosum | 24 | 1.487 | 0.569 |
| A. ramosum | A | A. ramosum | 27 | 1.266 | 0.388 |
| A. ramosum | B | A. ramosum | 29 | 0.742 | 0.158 |
| A. ramosum | B | A. ramosum | 17 | 0.501 | 0.106 |
| A. ramosum | B | A. ramosum | 26 | 0.883 | 0.134 |
| A. ramosum | B | A. ramosum | 31 | 0.139 | 0.006 |
| A. ramosum | B | A. ramosum | 32 | 0.062 | 0.011 |
| A. ramosum | B | A. ramosum | 34 | 0.819 | 0.229 |
| A. ramosum | B | A. ramosum | 32 | 1.142 | 0.423 |
| A. ramosum | B | A. ramosum | 28 | 0.096 | 0.032 |
| A. ramosum | B | A. ramosum | 32 | 0.416 | 0.107 |
| A. ramosum | B | A. ramosum | 31 | 1.106 | 0.291 |
| A. ramosum | A | B. erectus | 23 | 3.654 | 1.235 |
| A. ramosum | A | B. erectus | 11 | 3.138 | 1.988 |
| A. ramosum | A | B. erectus | 22 | 4.764 | 1.676 |
| A. ramosum | A | B. erectus | 26 | 1.87 | 1.438 |
| A. ramosum | A | B. erectus | 16 | 3.698 | 1.191 |
| A. ramosum | A | B. erectus | 26 | 9.038 | 3.674 |
| A. ramosum | A | B. erectus | 28 | 6.202 | 3.908 |
| A. ramosum | A | B. erectus | 26 | 4.369 | 2.23 |
| A. ramosum | A | B. erectus | 29 | 15.218 | 5.949 |
| A. ramosum | A | B. erectus | 35 | 3.707 | 1.773 |
| A. ramosum | B | B. erectus | 31 | 1.368 | 0.826 |
| A. ramosum | B | B. erectus | 23 | 0.84 | 0.316 |
| A. ramosum | B | B. erectus | 32 | 6.079 | 3.122 |
| A. ramosum | B | B. erectus | 21 | 2.95 | 0.894 |
| A. ramosum | B | B. erectus | 18 | 4.952 | 1.906 |
| A. ramosum | B | B. erectus | 18 | 2.974 | 1.48 |
| A. ramosum | B | B. erectus | 28 | 7.738 | 3.843 |
| A. ramosum | B | B. erectus | 26 | 8.091 | 4.218 |
| A. ramosum | B | B. erectus | 44 | 3.242 | 2.694 |
| A. ramosum | B | B. erectus | 27 | 3.578 | 2.323 |
| A. ramosum | A | B. pinnatum | 26 | 0.324 | 0.736 |
| A. ramosum | A | B. pinnatum | 11 | 0.435 | 0.483 |
| A. ramosum | A | B. pinnatum | 26 | 0.124 | 0.335 |
| A. ramosum | A | B. pinnatum | 23 | 0.237 | 0.286 |
| A. ramosum | A | B. pinnatum | 21 | 0.251 | 0.601 |
| A. ramosum | A | B. pinnatum | 17 | 0.325 | 0.569 |
| A. ramosum | A | B. pinnatum | 17 | 0.39 | 1.175 |
| A. ramosum | A | B. pinnatum | 21 | 1.279 | 1.297 |
| A. ramosum | A | B. pinnatum | 17 | 0.034 | 0.041 |
| A. ramosum | A | B. pinnatum | 13 | 0.526 | 0.659 |
| A. ramosum | B | B. pinnatum | 13 | 0.297 | 0.394 |
| A. ramosum | B | B. pinnatum | 22 | 1.032 | 1.042 |
| A. ramosum | B | B. pinnatum | 22 | 0.997 | 2.077 |
| A. ramosum | B | B. pinnatum | 27 | 1.024 | 1.197 |
| A. ramosum | B | B. pinnatum | 15 | 1.563 | 2.437 |
| A. ramosum | B | B. pinnatum | 19 | 1.527 | 1.282 |
| A. ramosum | B | B. pinnatum | 15 | 0.504 | 1.391 |
| A. ramosum | B | B. pinnatum | 21 | 2.521 | 2.571 |
| A. ramosum | B | B. pinnatum | 20 | 0.336 | 0.541 |
| A. ramosum | B | B. pinnatum | 9 | 2.304 | 1.221 |
| A. ramosum | A | I. salicina | 459 |  |  |
| A. ramosum | A | I. salicina | 573 | 0.1 | 0.3 |
| A. ramosum | A | I. salicina | 499 |  |  |
| A. ramosum | A | I. salicina | 522 |  |  |
| A. ramosum | A | I. salicina | 559 |  |  |
| A. ramosum | A | I. salicina | 459 |  |  |
| A. ramosum | A | I. salicina | 652 | 0.3 | 0.4 |
| A. ramosum | A | I. salicina | 413 |  |  |
| A. ramosum | A | I. salicina | 736 | 1.2 | 1.1 |
| A. ramosum | A | I. salicina | 258 |  |  |
| A. ramosum | B | I. salicina | 593 | 0.1 | 0.1 |
| A. ramosum | B | I. salicina | 647 |  |  |
| A. ramosum | B | I. salicina | 340 |  |  |
| A. ramosum | B | I. salicina | 651 |  |  |
| A. ramosum | B | I. salicina | 478 |  |  |
| A. ramosum | B | I. salicina | 678 | 0.1 | 0.2 |
| A. ramosum | B | I. salicina | 690 |  |  |
| A. ramosum | B | I. salicina | 317 |  |  |
| A. ramosum | B | I. salicina | 576 |  |  |
| A. ramosum | B | I. salicina | 220 |  |  |
| B. erectus | A | A. ramosum | 31 | 0.511 | 0.123 |
| B. erectus | A | A. ramosum | 27 | 0.635 | 0.164 |
| B. erectus | A | A. ramosum | 30 | 1.236 | 0.275 |
| B. erectus | A | A. ramosum | 32 | 0.805 | 0.175 |
| B. erectus | A | A. ramosum | 29 | 1.368 | 0.282 |
| B. erectus | A | A. ramosum | 22 | 0.841 | 0.149 |
| B. erectus | A | A. ramosum | 35 | 1.502 | 0.476 |
| B. erectus | A | A. ramosum | 31 | 0.922 | 0.434 |
| B. erectus | A | A. ramosum | 26 | 1.058 | 0.203 |
| B. erectus | A | A. ramosum | 27 | 0.48 | 0.236 |
| B. erectus | B | A. ramosum | 28 | 0.645 | 0.084 |
| B. erectus | B | A. ramosum | 29 | 0.883 | 0.164 |
| B. erectus | B | A. ramosum | 21 | 0.585 | 0.149 |
| B. erectus | B | A. ramosum | 37 | 0.29 | 0.074 |
| B. erectus | B | A. ramosum | 29 | 0.736 | 0.129 |
| B. erectus | B | A. ramosum | 25 | 0.517 | 0.199 |
| B. erectus | B | A. ramosum | 40 | 0.896 | 0.301 |
| B. erectus | B | A. ramosum | 27 | 0.819 | 0.195 |
| B. erectus | B | A. ramosum | 32 | 0.794 | 0.238 |
| B. erectus | B | A. ramosum | 35 | 0.794 | 0.221 |
| B. erectus | A | B. erectus | 11 | 1.555 | 0.509 |
| B. erectus | A | B. erectus | 22 | 4.121 | 3.837 |
| B. erectus | A | B. erectus | 28 |  |  |
| B. erectus | A | B. erectus | 21 | 2.123 | 1.271 |
| B. erectus | A | B. erectus | 12 | 5.009 | 1.969 |
| B. erectus | A | B. erectus | 28 | 2.052 | 1.255 |
| B. erectus | A | B. erectus | 27 | 2.128 | 1.087 |
| B. erectus | A | B. erectus | 34 | 3.751 | 3.401 |
| B. erectus | A | B. erectus | 35 | 3.628 | 1.402 |
| B. erectus | A | B. erectus | 24 | 2.404 | 1.777 |
| B. erectus | B | B. erectus | 26 | 1.117 | 0.867 |
| B. erectus | B | B. erectus | 17 | 1.073 | 0.556 |
| B. erectus | B | B. erectus | 21 | 0.277 | 0.256 |
| B. erectus | B | B. erectus | 21 | 0.867 | 0.377 |
| B. erectus | B | B. erectus | 20 | 1.073 | 0.618 |
| B. erectus | B | B. erectus | 33 | 0.809 | 0.471 |
| B. erectus | B | B. erectus | 28 | 1.198 | 0.692 |
| B. erectus | B | B. erectus | 24 | 2.69 | 1.788 |
| B. erectus | B | B. erectus | 27 | 1.268 | 0.782 |
| B. erectus | B | B. erectus | 30 | 1.556 | 1.121 |
| B. erectus | A | B. pinnatum | 15 | 0.265 | 0.249 |
| B. erectus | A | B. pinnatum | 16 | 0.257 | 0.74 |
| B. erectus | A | B. pinnatum | 19 | 0.215 | 0.37 |
| B. erectus | A | B. pinnatum | 12 | 0.367 | 1.35 |
| B. erectus | A | B. pinnatum | 16 | 1.232 | 1.054 |
| B. erectus | A | B. pinnatum | 12 | 0.735 | 2.854 |
| B. erectus | A | B. pinnatum | 17 | 0.238 | 0.489 |
| B. erectus | A | B. pinnatum | 10 | 0.83 | 0.985 |
| B. erectus | A | B. pinnatum | 18 | 0.497 | 0.618 |
| B. erectus | A | B. pinnatum | 11 | 0.055 | 0.122 |
| B. erectus | B | B. pinnatum | 25 | 0.173 | 0.355 |
| B. erectus | B | B. pinnatum | 15 | 0.562 | 0.257 |
| B. erectus | B | B. pinnatum | 21 | 0.566 | 1.073 |
| B. erectus | B | B. pinnatum | 24 | 0.497 | 1.137 |
| B. erectus | B | B. pinnatum | 22 | 0.64 | 1.165 |
| B. erectus | B | B. pinnatum | 10 | 0.688 | 0.757 |
| B. erectus | B | B. pinnatum | 14 | 0.856 | 1.252 |
| B. erectus | B | B. pinnatum | 15 | 0.824 | 0.801 |
| B. erectus | B | B. pinnatum | 17 | 0.727 | 0.59 |
| B. erectus | B | B. pinnatum | 15 | 0.23 | 0.468 |
| B. erectus | A | I. salicina | 588 | 0.2 | 0.4 |
| B. erectus | A | I. salicina | 571 | 0.1 | 0.1 |
| B. erectus | A | I. salicina | 393 |  |  |
| B. erectus | A | I. salicina | 477 |  |  |
| B. erectus | A | I. salicina | 597 | 0.2 | 0.4 |
| B. erectus | A | I. salicina | 634 | 0.2 | 0.2 |
| B. erectus | A | I. salicina | 363 |  |  |
| B. erectus | A | I. salicina | 284 |  |  |
| B. erectus | A | I. salicina | 551 |  |  |
| B. erectus | A | I. salicina | 351 |  |  |
| B. erectus | B | I. salicina | 644 |  |  |
| B. erectus | B | I. salicina | 291 | 0.1 | 0.2 |
| B. erectus | B | I. salicina | 387 |  |  |
| B. erectus | B | I. salicina | 651 |  |  |
| B. erectus | B | I. salicina | 732 |  |  |
| B. erectus | B | I. salicina | 609 | 0.1 | 0.1 |
| B. erectus | B | I. salicina | 221 |  |  |
| B. erectus | B | I. salicina | 404 |  |  |
| B. erectus | B | I. salicina | 807 | 0.1 | 0.1 |
| B. erectus | B | I. salicina | 488 |  |  |
| B. pinnatum | A | A. ramosum | 20 | 0.035 | 0.009 |
| B. pinnatum | A | A. ramosum | 15 | 0.225 | 0.057 |
| B. pinnatum | A | A. ramosum | 35 | 0.361 | 0.1 |
| B. pinnatum | A | A. ramosum | 23 | 0.943 | 0.227 |
| B. pinnatum | A | A. ramosum | 24 | 1.038 | 0.426 |
| B. pinnatum | A | A. ramosum | 30 | 1.086 | 0.462 |
| B. pinnatum | A | A. ramosum | 31 | 0.58 | 0.294 |
| B. pinnatum | A | A. ramosum | 31 | 0.781 | 0.29 |
| B. pinnatum | A | A. ramosum | 27 | 1.143 | 0.285 |
| B. pinnatum | A | A. ramosum | 31 | 1.21 | 0.196 |
| B. pinnatum | B | A. ramosum | 38 | 0.847 | 0.16 |
| B. pinnatum | B | A. ramosum | 20 | 0.769 | 0.176 |
| B. pinnatum | B | A. ramosum | 35 | 0.051 | 0.011 |
| B. pinnatum | B | A. ramosum | 22 | 0.511 | 0.181 |
| B. pinnatum | B | A. ramosum | 29 | 0.12 | 0.007 |
| B. pinnatum | B | A. ramosum | 33 | 0.171 | 0.063 |
| B. pinnatum | B | A. ramosum | 32 | 1.145 | 0.368 |
| B. pinnatum | B | A. ramosum | 36 | 0.741 | 0.365 |
| B. pinnatum | B | A. ramosum | 28 | 0.287 | 0.119 |
| B. pinnatum | B | A. ramosum | 34 | 1.135 | 0.333 |
| B. pinnatum | A | B. erectus | 17 | 1.133 | 0.649 |
| B. pinnatum | A | B. erectus | 9 | 0.444 | 0.169 |
| B. pinnatum | A | B. erectus | 17 | 0.589 | 0.266 |
| B. pinnatum | A | B. erectus | 17 | 7.743 | 5.833 |
| B. pinnatum | A | B. erectus | 15 | 1.704 | 1.37 |
| B. pinnatum | A | B. erectus | 9 | 5.977 | 4.895 |
| B. pinnatum | A | B. erectus | 19 | 2.483 | 0.658 |
| B. pinnatum | A | B. erectus | 30 | 1.844 | 1.214 |
| B. pinnatum | A | B. erectus | 30 | 2.145 | 1.527 |
| B. pinnatum | A | B. erectus | 33 | 5.867 | 2.427 |
| B. pinnatum | B | B. erectus | 28 | 1.252 | 0.301 |
| B. pinnatum | B | B. erectus | 22 | 2.635 | 0.828 |
| B. pinnatum | B | B. erectus | 19 | 3.386 | 1.939 |
| B. pinnatum | B | B. erectus | 24 | 2.555 | 2.049 |
| B. pinnatum | B | B. erectus | 12 | 7.259 | 3.39 |
| B. pinnatum | B | B. erectus | 26 | 2.439 | 0.905 |
| B. pinnatum | B | B. erectus | 28 | 2.811 | 1.604 |
| B. pinnatum | B | B. erectus | 33 | 5.348 | 3.659 |
| B. pinnatum | B | B. erectus | 18 | 1.566 | 0.877 |
| B. pinnatum | B | B. erectus | 25 | 2.854 | 1.595 |
| B. pinnatum | A | B. pinnatum | 26 | 0.128 | 0.261 |
| B. pinnatum | A | B. pinnatum | 23 | 0.141 | 0.286 |
| B. pinnatum | A | B. pinnatum | 18 | 1.069 | 1.04 |
| B. pinnatum | A | B. pinnatum | 16 | 0.435 | 1.1 |
| B. pinnatum | A | B. pinnatum | 15 | 0.313 | 0.43 |
| B. pinnatum | A | B. pinnatum | 11 | 0.29 | 0.568 |
| B. pinnatum | A | B. pinnatum | 16 | 1.093 | 1.695 |
| B. pinnatum | A | B. pinnatum | 11 | 0.093 | 0.221 |
| B. pinnatum | A | B. pinnatum | 14 | 0.14 | 0.533 |
| B. pinnatum | A | B. pinnatum | 11 | 0.384 | 0.543 |
| B. pinnatum | B | B. pinnatum | 28 | 0.502 | 0.252 |
| B. pinnatum | B | B. pinnatum | 17 | 0.715 | 0.365 |
| B. pinnatum | B | B. pinnatum | 29 | 0.66 | 0.762 |
| B. pinnatum | B | B. pinnatum | 17 | 0.866 | 1.238 |
| B. pinnatum | B | B. pinnatum | 18 | 0.459 | 0.263 |
| B. pinnatum | B | B. pinnatum | 9 | 0.484 | 0.896 |
| B. pinnatum | B | B. pinnatum | 14 | 1.097 | 1.661 |
| B. pinnatum | B | B. pinnatum | 13 | 2.279 | 0.921 |
| B. pinnatum | B | B. pinnatum | 7 | 0.121 | 0.309 |
| B. pinnatum | B | B. pinnatum | 9 | 0.71 | 1.102 |
| B. pinnatum | A | I. salicina | 594 | 0.7 | 2.4 |
| B. pinnatum | A | I. salicina | 560 | 0.2 | 0.2 |
| B. pinnatum | A | I. salicina | 712 |  |  |
| B. pinnatum | A | I. salicina | 524 |  |  |
| B. pinnatum | A | I. salicina | 629 |  |  |
| B. pinnatum | A | I. salicina | 527 |  |  |
| B. pinnatum | A | I. salicina | 430 | 0.6 | 0.5 |
| B. pinnatum | A | I. salicina | 449 |  |  |
| B. pinnatum | A | I. salicina | 369 |  |  |
| B. pinnatum | A | I. salicina | 461 |  |  |
| B. pinnatum | B | I. salicina | 473 | 0.1 | 0.2 |
| B. pinnatum | B | I. salicina | 615 | 0.1 | 0.2 |
| B. pinnatum | B | I. salicina | 479 | 0.3 | 0.5 |
| B. pinnatum | B | I. salicina | 805 |  |  |
| B. pinnatum | B | I. salicina | 461 |  |  |
| B. pinnatum | B | I. salicina | 479 |  |  |
| B. pinnatum | B | I. salicina | 289 |  |  |
| B. pinnatum | B | I. salicina | 368 |  |  |
| B. pinnatum | B | I. salicina | 279 |  |  |
| B. pinnatum | B | I. salicina | 327 | 0.1 | 0.1 |
| Control | A | A. ramosum | 25 | 0.348 | 0.068 |
| Control | A | A. ramosum | 5 | 0.747 | 0.187 |
| Control | A | A. ramosum | 35 | 1.087 | 0.276 |
| Control | A | A. ramosum | 25 | 0.837 | 0.267 |
| Control | A | A. ramosum | 23 | 0.627 | 0.15 |
| Control | A | A. ramosum | 30 | 0.962 | 0.271 |
| Control | A | A. ramosum | 32 | 0.995 | 0.398 |
| Control | A | A. ramosum | 26 | 0.575 | 0.242 |
| Control | A | A. ramosum | 37 | 0.873 | 0.179 |
| Control | A | A. ramosum | 29 | 0.79 | 0.23 |
| Control | B | A. ramosum | 28 | 0.698 | 0.148 |
| Control | B | A. ramosum | 26 | 0.909 | 0.151 |
| Control | B | A. ramosum | 36 | 0.895 | 0.221 |
| Control | B | A. ramosum | 31 | 1.187 | 0.344 |
| Control | B | A. ramosum | 33 | 0.887 | 0.172 |
| Control | B | A. ramosum | 34 | 1.412 | 0.484 |
| Control | B | A. ramosum | 39 | 0.43 | 0.121 |
| Control | B | A. ramosum | 35 | 0.118 | 0.64 |
| Control | B | A. ramosum | 39 | 0.498 | 0.143 |
| Control | B | A. ramosum | 30 | 1.052 | 0.48 |
| Control | A | B. erectus | 25 | 1.307 | 0.834 |
| Control | A | B. erectus | 27 | 1.709 | 0.606 |
| Control | A | B. erectus | 26 | 2.479 | 0.887 |
| Control | A | B. erectus | 20 | 3.233 | 2.507 |
| Control | A | B. erectus | 18 | 3.885 | 2.24 |
| Control | A | B. erectus | 25 | 1.987 | 1.045 |
| Control | A | B. erectus | 27 | 4.407 | 1.799 |
| Control | A | B. erectus | 34 | 5.946 | 3.368 |
| Control | A | B. erectus | 22 | 4.815 | 3.361 |
| Control | A | B. erectus | 26 | 7.379 | 3.63 |
| Control | B | B. erectus | 24 | 3.062 | 1.992 |
| Control | B | B. erectus | 22 | 2.261 | 1.281 |
| Control | B | B. erectus | 22 | 3.99 | 2.886 |
| Control | B | B. erectus | 26 | 4.692 | 1.331 |
| Control | B | B. erectus | 16 | 11.187 | 4.436 |
| Control | B | B. erectus | 22 | 13.205 | 6.575 |
| Control | B | B. erectus | 29 | 11.85 | 5.569 |
| Control | B | B. erectus | 27 | 5.405 | 3.257 |
| Control | B | B. erectus | 37 | 4.029 | 1.814 |
| Control | B | B. erectus | 31 | 6.835 | 2.589 |
| Control | A | B. pinnatum | 24 | 0.499 | 1.073 |
| Control | A | B. pinnatum | 30 | 0.86 | 1.87 |
| Control | A | B. pinnatum | 21 | 0.766 | 2.104 |
| Control | A | B. pinnatum | 22 | 0.555 | 1.035 |
| Control | A | B. pinnatum | 12 | 0.449 | 0.463 |
| Control | A | B. pinnatum | 5 | 1.313 | 1.089 |
| Control | A | B. pinnatum | 13 | 0.151 | 0.247 |
| Control | A | B. pinnatum | 16 | 0.346 | 0.204 |
| Control | A | B. pinnatum | 9 | 0.775 | 1.572 |
| Control | A | B. pinnatum | 4 | 0.526 | 0.79 |
| Control | B | B. pinnatum | 21 | 0.295 | 0.664 |
| Control | B | B. pinnatum | 22 | 1.299 | 0.937 |
| Control | B | B. pinnatum | 14 | 0.233 | 0.471 |
| Control | B | B. pinnatum | 20 | 0.538 | 0.976 |
| Control | B | B. pinnatum | 15 | 0.53 | 0.734 |
| Control | B | B. pinnatum | 12 | 0.198 | 0.616 |
| Control | B | B. pinnatum | 14 | 0.86 | 0.944 |
| Control | B | B. pinnatum | 18 | 0.49 | 1.05 |
| Control | B | B. pinnatum | 13 | 0.083 | 0.133 |
| Control | B | B. pinnatum | 15 | 0.535 | 0.723 |
| Control | A | I. salicina | 427 | 0.1 | 0.2 |
| Control | A | I. salicina | 500 | 0.1 | 0.1 |
| Control | A | I. salicina | 301 | 0.1 | 0.1 |
| Control | A | I. salicina | 354 | 0.6 | 0.8 |
| Control | A | I. salicina | 383 |  |  |
| Control | A | I. salicina | 504 | 0.7 | 0.7 |
| Control | A | I. salicina | 485 | 0.3 | 0.3 |
| Control | A | I. salicina | 688 | 0.3 | 0.4 |
| Control | A | I. salicina | 274 |  |  |
| Control | A | I. salicina | 547 | 0.2 | 0.5 |
| Control | B | I. salicina | 523 |  |  |
| Control | B | I. salicina | 508 |  |  |
| Control | B | I. salicina | 460 |  |  |
| Control | B | I. salicina | 651 |  |  |
| Control | B | I. salicina | 472 |  |  |
| Control | B | I. salicina | 614 | 0.3 | 0.3 |
| Control | B | I. salicina | 271 | 0.3 | 0.4 |
| Control | B | I. salicina | 564 |  |  |
| Control | B | I. salicina | 640 |  |  |
| Control | B | I. salicina | 521 |  |  |
| I. salicina | A | A. ramosum | 25 | 1.136 | 0.217 |
| I. salicina | A | A. ramosum | 22 | 1.236 | 0.279 |
| I. salicina | A | A. ramosum | 31 | 0.708 | 0.186 |
| I. salicina | A | A. ramosum | 29 | 0.514 | 0.158 |
| I. salicina | A | A. ramosum | 22 | 0.766 | 0.17 |
| I. salicina | A | A. ramosum | 29 | 0.646 | 0.101 |
| I. salicina | A | A. ramosum | 35 | 1.657 | 0.839 |
| I. salicina | A | A. ramosum | 28 | 0.691 | 0.157 |
| I. salicina | A | A. ramosum | 34 | 1.351 | 0.49 |
| I. salicina | A | A. ramosum | 33 | 1.503 | 0.384 |
| I. salicina | B | A. ramosum | 32 | 0.745 | 0.139 |
| I. salicina | B | A. ramosum | 32 | 0.575 | 0.124 |
| I. salicina | B | A. ramosum | 26 | 0.789 | 0.157 |
| I. salicina | B | A. ramosum | 38 | 1.291 | 0.393 |
| I. salicina | B | A. ramosum | 31 | 0.722 | 0.164 |
| I. salicina | B | A. ramosum | 37 | 0.753 | 0.409 |
| I. salicina | B | A. ramosum | 31 | 1.112 | 0.231 |
| I. salicina | B | A. ramosum | 33 | 1.58 | 0.715 |
| I. salicina | B | A. ramosum | 27 | 1.433 | 0.275 |
| I. salicina | B | A. ramosum | 30 | 0.43 | 0.108 |
| I. salicina | A | B. erectus | 28 | 1.515 | 0.34 |
| I. salicina | A | B. erectus | 35 | 1.422 | 0.766 |
| I. salicina | A | B. erectus | 25 | 3.16 | 2.543 |
| I. salicina | A | B. erectus | 21 | 2.848 | 2.055 |
| I. salicina | A | B. erectus | 16 | 3.352 | 1.4 |
| I. salicina | A | B. erectus | 25 | 3.728 | 1.712 |
| I. salicina | A | B. erectus | 11 | 3.37 | 1.062 |
| I. salicina | A | B. erectus | 17 | 2.718 | 1.009 |
| I. salicina | A | B. erectus | 27 | 7.784 | 4.249 |
| I. salicina | A | B. erectus | 15 | 0.822 | 1.017 |
| I. salicina | B | B. erectus | 24 | 0.832 | 0.223 |
| I. salicina | B | B. erectus | 26 | 2.205 | 0.911 |
| I. salicina | B | B. erectus | 19 | 5.901 | 2.335 |
| I. salicina | B | B. erectus | 31 | 11.43 | 4.167 |
| I. salicina | B | B. erectus | 12 | 5.132 | 2.977 |
| I. salicina | B | B. erectus | 9 | 5.135 | 2.135 |
| I. salicina | B | B. erectus | 31 | 5.908 | 2.782 |
| I. salicina | B | B. erectus | 34 | 11.033 | 4.902 |
| I. salicina | B | B. erectus | 33 | 2.774 | 1.096 |
| I. salicina | B | B. erectus | 31 | 6.763 | 1.867 |
| I. salicina | A | B. pinnatum | 18 | 0.99 | 0.714 |
| I. salicina | A | B. pinnatum | 23 | 0.239 | 0.856 |
| I. salicina | A | B. pinnatum | 21 | 0.96 | 1.673 |
| I. salicina | A | B. pinnatum | 24 | 0.693 | 0.845 |
| I. salicina | A | B. pinnatum | 17 | 0.864 | 1.324 |
| I. salicina | A | B. pinnatum | 21 | 0.306 | 0.767 |
| I. salicina | A | B. pinnatum | 19 | 3.675 | 2.274 |
| I. salicina | A | B. pinnatum | 9 | 0.381 | 1.467 |
| I. salicina | A | B. pinnatum | 16 | 0.817 | 1.666 |
| I. salicina | A | B. pinnatum | 12 | 0.872 | 0.731 |
| I. salicina | B | B. pinnatum | 17 | 0.272 | 1.239 |
| I. salicina | B | B. pinnatum | 16 | 0.35 | 0.744 |
| I. salicina | B | B. pinnatum | 21 | 1.451 | 1.912 |
| I. salicina | B | B. pinnatum | 24 | 0.578 | 1.157 |
| I. salicina | B | B. pinnatum | 20 | 0.672 | 1.432 |
| I. salicina | B | B. pinnatum | 23 | 0.744 | 0.811 |
| I. salicina | B | B. pinnatum | 15 | 0.677 | 1.765 |
| I. salicina | B | B. pinnatum | 11 | 2.455 | 1.477 |
| I. salicina | B | B. pinnatum | 9 | 1.079 | 1.097 |
| I. salicina | B | B. pinnatum | 8 | 1.423 | 1.386 |
| I. salicina | A | I. salicina | 439 |  |  |
| I. salicina | A | I. salicina | 379 | 0.1 | 0.1 |
| I. salicina | A | I. salicina | 485 |  |  |
| I. salicina | A | I. salicina | 516 |  |  |
| I. salicina | A | I. salicina | 378 |  |  |
| I. salicina | A | I. salicina | 342 |  |  |
| I. salicina | A | I. salicina | 144 |  |  |
| I. salicina | A | I. salicina | 486 |  |  |
| I. salicina | A | I. salicina | 274 |  |  |
| I. salicina | A | I. salicina | 270 | 0.2 | 0.2 |
| I. salicina | B | I. salicina | 417 |  |  |
| I. salicina | B | I. salicina | 410 |  |  |
| I. salicina | B | I. salicina | 601 |  |  |
| I. salicina | B | I. salicina | 567 |  |  |
| I. salicina | B | I. salicina | 316 |  |  |
| I. salicina | B | I. salicina | 0 |  |  |
| I. salicina | B | I. salicina | 99 | 0.3 | 0.2 |
| I. salicina | B | I. salicina | 0 |  |  |
| I. salicina | B | I. salicina | 525 |  |  |
| I. salicina | B | I. salicina | 379 |  |  |
| Mix | A | A. ramosum | 25 | 0.704 | 0.146 |
| Mix | A | A. ramosum | 29 | 0.819 | 0.193 |
| Mix | A | A. ramosum | 24 | 1.079 | 0.252 |
| Mix | A | A. ramosum | 32 | 0.763 | 0.131 |
| Mix | A | A. ramosum | 25 | 0.761 | 0.194 |
| Mix | A | A. ramosum | 24 | 1.184 | 0.379 |
| Mix | A | A. ramosum | 33 | 1.317 | 0.327 |
| Mix | A | A. ramosum | 21 | 0.778 | 0.202 |
| Mix | A | A. ramosum | 35 | 1.198 | 0.312 |
| Mix | A | A. ramosum | 27 |  |  |
| Mix | B | A. ramosum | 30 | 0.263 | 0.059 |
| Mix | B | A. ramosum | 35 | 1.231 | 0.273 |
| Mix | B | A. ramosum | 44 | 0.32 | 0.81 |
| Mix | B | A. ramosum | 35 | 0.431 | 0.155 |
| Mix | B | A. ramosum | 38 | 0.1 | 0.294 |
| Mix | B | A. ramosum | 32 | 1.129 | 0.231 |
| Mix | B | A. ramosum | 46 | 1.049 | 0.255 |
| Mix | B | A. ramosum | 36 | 1 | 0.459 |
| Mix | B | A. ramosum | 33 | 0.133 | 0.009 |
| Mix | B | A. ramosum | 27 | 0.971 | 0.352 |
| Mix | A | B. erectus | 33 | 2.971 | 1.658 |
| Mix | A | B. erectus | 29 | 2.849 | 3.029 |
| Mix | A | B. erectus | 27 | 1.413 | 0.766 |
| Mix | A | B. erectus | 31 | 2.695 | 2.651 |
| Mix | A | B. erectus | 16 | 4.495 | 2.432 |
| Mix | A | B. erectus | 22 | 4.554 | 1.783 |
| Mix | A | B. erectus | 29 | 5.467 | 3.407 |
| Mix | A | B. erectus | 22 | 2.539 | 1.05 |
| Mix | A | B. erectus | 20 | 2.736 | 2.332 |
| Mix | A | B. erectus | 31 | 2.698 | 1.333 |
| Mix | B | B. erectus | 34 | 1.05 | 0.331 |
| Mix | B | B. erectus | 22 | 2.131 | 1.516 |
| Mix | B | B. erectus | 21 | 2.654 | 1.095 |
| Mix | B | B. erectus | 8 | 1.536 | 1.293 |
| Mix | B | B. erectus | 15 | 2.467 | 0.648 |
| Mix | B | B. erectus | 16 | 5.477 | 3.4 |
| Mix | B | B. erectus | 28 | 4.644 | 2.006 |
| Mix | B | B. erectus | 8 | 2.934 | 1.664 |
| Mix | B | B. erectus | 25 | 4.731 | 2.621 |
| Mix | B | B. erectus | 34 | 2.037 | 0.952 |
| Mix | A | B. pinnatum | 25 | 0.425 | 1.009 |
| Mix | A | B. pinnatum | 25 | 0.055 | 1.093 |
| Mix | A | B. pinnatum | 22 | 0.463 | 1.2 |
| Mix | A | B. pinnatum | 16 | 0.328 | 0.651 |
| Mix | A | B. pinnatum | 15 | 1.042 | 0.849 |
| Mix | A | B. pinnatum | 16 | 0.059 | 0.093 |
| Mix | A | B. pinnatum | 15 | 0.163 | 0.25 |
| Mix | A | B. pinnatum | 13 | 0.564 | 0.824 |
| Mix | A | B. pinnatum | 7 | 0.061 | 0.199 |
| Mix | A | B. pinnatum | 19 | 0.012 | 0.015 |
| Mix | B | B. pinnatum | 21 | 0.131 | 0.428 |
| Mix | B | B. pinnatum | 21 | 0.431 | 0.235 |
| Mix | B | B. pinnatum | 21 | 3.12 | 2.559 |
| Mix | B | B. pinnatum | 16 | 1.585 | 2.184 |
| Mix | B | B. pinnatum | 24 | 1.349 | 0.512 |
| Mix | B | B. pinnatum | 18 | 1.052 | 0.805 |
| Mix | B | B. pinnatum | 13 | 1.318 | 1.718 |
| Mix | B | B. pinnatum | 21 | 0.691 | 0.908 |
| Mix | B | B. pinnatum | 19 | 0.637 | 0.759 |
| Mix | B | B. pinnatum | 18 | 0.51 | 0.588 |
| Mix | A | I. salicina | 274 |  |  |
| Mix | A | I. salicina | 693 |  |  |
| Mix | A | I. salicina | 2 |  |  |
| Mix | A | I. salicina | 369 |  |  |
| Mix | A | I. salicina | 235 | 0.1 | 0.1 |
| Mix | A | I. salicina | 358 | 0.6 | 0.4 |
| Mix | A | I. salicina | 214 | 0.1 | 0.1 |
| Mix | A | I. salicina | 270 | 0.6 | 0.7 |
| Mix | A | I. salicina | 573 |  |  |
| Mix | A | I. salicina | 411 |  |  |
| Mix | B | I. salicina | 664 | 0.1 | 0.1 |
| Mix | B | I. salicina | 628 | 0.1 | 0.1 |
| Mix | B | I. salicina | 670 |  |  |
| Mix | B | I. salicina | 716 |  |  |
| Mix | B | I. salicina | 796 |  |  |
| Mix | B | I. salicina | 566 |  |  |
| Mix | B | I. salicina | 673 |  |  |
| Mix | B | I. salicina | 567 |  |  |
| Mix | B | I. salicina | 579 |  |  |
| Mix | B | I. salicina | 691 |  |  |
